# Defining the Role of ATP Hydrolysis in Mitotic Segregation of Bacterial Plasmids

**DOI:** 10.1371/journal.pgen.1003956

**Published:** 2013-12-19

**Authors:** Yoan Ah-Seng, Jérôme Rech, David Lane, Jean-Yves Bouet

**Affiliations:** Laboratoire de Microbiologie et Génétique Moléculaires, Centre National de Recherche Scientifique et l'Université Paul Sabatier, Toulouse, France; University of Geneva Medical School, Switzerland

## Abstract

Hydrolysis of ATP by partition ATPases, although considered a key step in the segregation mechanism that assures stable inheritance of plasmids, is intrinsically very weak. The cognate centromere-binding protein (CBP), together with DNA, stimulates the ATPase to hydrolyse ATP and to undertake the relocation that incites plasmid movement, apparently confirming the need for hydrolysis in partition. However, ATP-binding alone changes ATPase conformation and properties, making it difficult to rigorously distinguish the substrate and cofactor roles of ATP *in vivo*. We had shown that mutation of arginines R36 and R42 in the F plasmid CBP, SopB, reduces stimulation of SopA-catalyzed ATP hydrolysis without changing SopA-SopB affinity, suggesting the role of hydrolysis could be analyzed using SopA with normal conformational responses to ATP. Here, we report that strongly reducing SopB-mediated stimulation of ATP hydrolysis results in only slight destabilization of mini-F, although the instability, as well as an increase in mini-F clustering, is proportional to the ATPase deficit. Unexpectedly, the reduced stimulation also increased the frequency of SopA relocation over the nucleoid. The increase was due to drastic shortening of the period spent by SopA at nucleoid ends; average speed of migration *per se* was unchanged. Reduced ATP hydrolysis was also associated with pronounced deviations in positioning of mini-F, though time-averaged positions changed only modestly. Thus, by specifically targeting SopB-stimulated ATP hydrolysis our study reveals that even at levels of ATPase which reduce the efficiency of splitting clusters and the constancy of plasmid positioning, SopB still activates SopA mobility and plasmid positioning, and sustains near wild type levels of plasmid stability.

## Author Summary

Genes enabling bacteria to survive and thrive in challenging environments are very often found on small, non-essential DNA molecules called plasmids. Many plasmids are naturally present in the cell in very few copies and so risk being lost from one of the daughter cells upon division. These plasmids elaborate a partition system, functionally similar to mitosis, which assures their faithful inheritance. Chromosomes also generally possess such systems. We know that partition systems involve two proteins, that one (B) stimulates the other (A) to hydrolyse ATP, and that upon binding to A protein ATP confers properties needed for partition. ATP's double action, as hydrolysis substrate and cofactor, complicates definition of its role in the mechanism. The novelty of our approach lies in use of B protein mutants that do not stimulate hydrolysis. Our results reveal that the major function of ATP hydrolysis is not to displace plasmid molecules to their positions in each cell half, as generally thought, but to split initial sibling plasmid pairs and prevent their reforming. This study is the first to dissect ATPase activity *in vivo* using normal A-protein ATPase, and so opens a new avenue to exploration of the mechanisms that ensure plasmid and chromosome inheritance.

## Introduction

Partition, the elementary form of mitosis that segregates sibling replicons in bacteria, depends critically on replicon-specific NTPases. These proteins, generically termed ParA, contact their replicons by interacting with the cognate ParB protein bound to a set of short sequence repeats (*parS*) that serves as the centromere. The interaction provokes dynamic behaviour of ParA which somehow directs replicon copies to the new cells-to-be.

By far the most common type of partition NTPase is characterized by a variant ATP-binding Walker A box motif [1], [2] (reviewed by Vecchiarelli *et al.* [3]). These ParAs are specified by most low copy number plasmids and are the only type encoded by sequenced chromosomes. Mutation of conserved ATP interaction motifs in ParA proteins leads to frequent mis-localization and segregation failure, particularly of low copy-number plasmids [4]–[8], leaving no doubt that ATP is essential to partition.

Just how ATP is involved is harder to define. The ATP hydrolysis activity of Walker-box ParA proteins is intrinsically very weak but is synergistically stimulated *in vitro* by cognate ParB proteins and by DNA [9]–[11], particularly when the latter includes the centromere [12]. Likewise, the ability of these ParA proteins to migrate *en masse* from end to end of the nucleoid a few times per cell cycle, presumed to be relevant to plasmid segregation [6], [13], is seen only in the presence of the cognate ParB and *parS*. These observations, together with the parallel effects of ATPase mutations on partition and ParA dynamics and the correlated movements of ParA and its plasmid [6], [13]–[16], argue for the involvement of ATP hydrolysis in the partition mechanism. On the other hand, binding of ATP without hydrolysis changes the conformation of ParA proteins [7], [17] and alters their properties: it facilitates dimerization of ParA [18]–20 and it enables dimers to bind to non-specific DNA [20]–[23] as well as to each other to form polymeric assemblies [4], [14], [15], [21], [22], [24], [25]. Thus, even in the case of partition-defective mutants that demonstrably bind but fail to hydrolyse ATP, it has not been possible to exclude conformational effects as the key reason for the functional deficiency.

We have recently reported that in the case of the Sop partition system of the *Escherichia coli* plasmid F, mutation of two arginine residues in the SopB N-terminus, neither of which significantly affects affinity for SopA, reduces stimulation of SopA-catalyzed ATP hydrolysis *in vitro* [12]. The mutants are interesting for two reasons. One is mechanistic: the characteristics of R36 and R42 mutants are consistent with the former acting as an arginine-finger [26], inserting itself into the ATP-binding pocket of SopA to facilitate processing of the transition state, and the latter serving to optimize positioning of the finger, as previously suggested for analogous mutations in the ParG partition protein of plasmid TP228 [4].

The other reason is the delegation of responsibility for modulating ATP hydrolysis to a physically distinct partner protein. It should be possible to use the SopB arginine-finger mutants to reduce ATP hydrolysis by wild type SopA protein whose conformational responses to ATP binding are normal, and thus to distinguish the substrate and cofactor roles of ATP for any activity in which SopA is involved. We report here that SopB-mediated stimulation of ATP hydrolysis by SopA is needed to restrain oscillatory movement of SopA, to stabilize plasmid positioning and to limit plasmid clustering.

## Results

In our previous *in vitro* study [12], we found hydrolysis of ATP by SopA to be stimulated three-fold by SopB alone and a further 16- and 45-fold when non-specific and *sopC*-containing DNA, respectively, were also present. SopB mutation R36A reduced ATPase stimulation by about 20-fold, R36K by seven-fold and R42A by six-fold. Such decreases, if reproduced *in vivo*, would be expected to provoke frequent loss of mini-F from dividing cells.

### Mini-F stability

**Figure 1:**
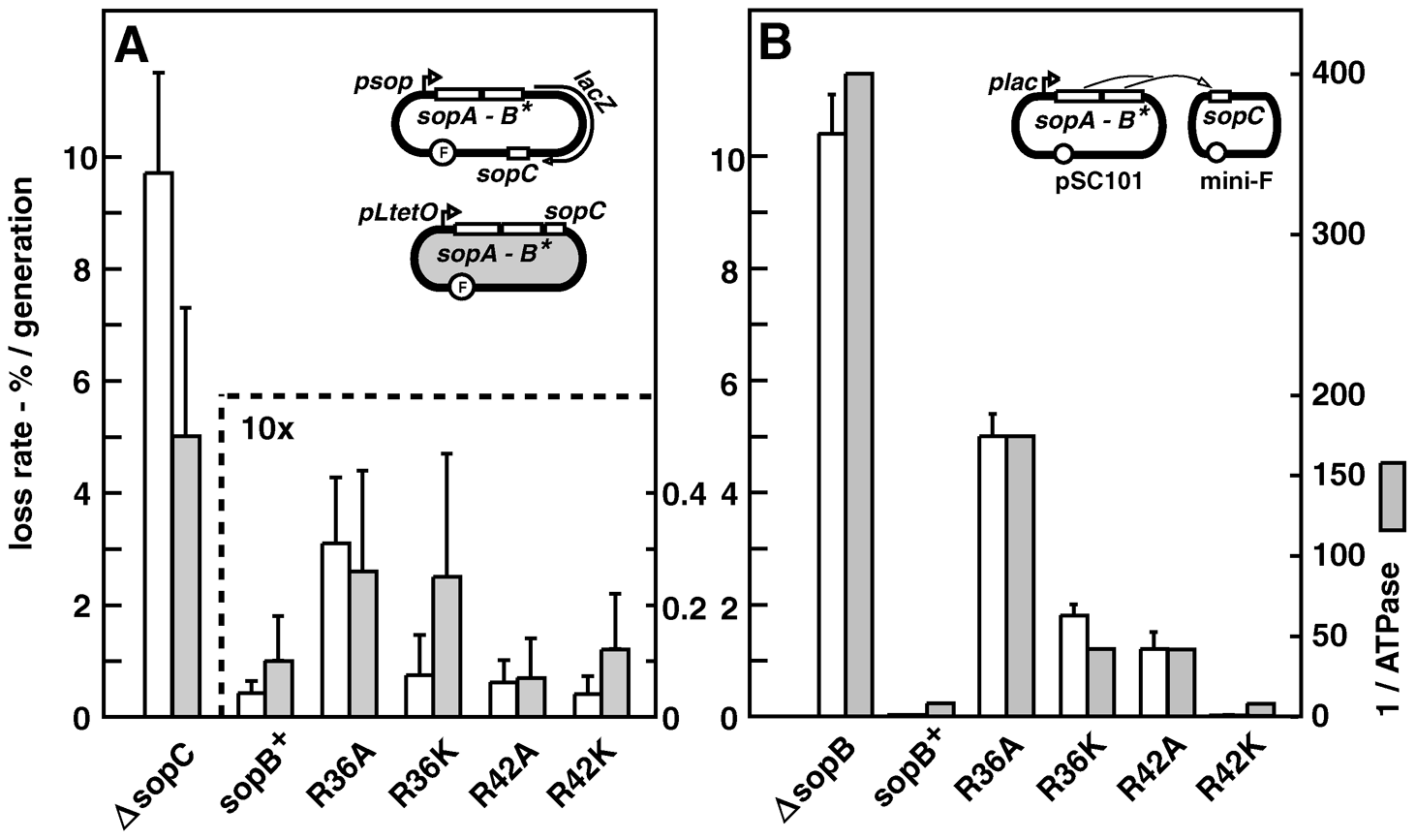
Effect of SopB R36 and R42 mutations on mini-F stability. **A**. *sopAB* operon and *sopC* in natural configuration (*cis*; see sketches): white bars - strain DLT1900 carrying pDAG218 and derivatives, grown at 30°C in minimal-glycerol-leucine medium (t∼160 min); shaded bars - strain DH10B carrying pDAG173 and derivatives, grown in minimal-glycerol-Casamino acids medium (t∼80 min). Error bars are standard deviations of ≥3 loss-rate assays. Note 10-fold scale change for *sopC* ^+^ plasmids. **B**. *sopAB* and *sopC* on separate plasmids, pAM138 and mini-F respectively (*trans*), in strain MC1061 grown in minimal-glycerol-leucine medium: white bars – loss rates. Shaded bars show ATPase deficits as the inverse of ATPase stimulation by each SopB *in vitro*, determined previously [12].

We substituted the R36 and R42 alleles for wild type *sopB* in two normally stable mini-F plasmids. In one, pDAG218, a promoterless *lacZ* gene is inserted between *sopB* and the *sopC* centromere, allowing direct monitoring of plasmid loss and of transcription from the natural, autoregulated *sop* promoter. Assays of plasmid retention during growth in non-selective minimal-glycerol medium (Figure 1A) showed the mutants to be surprisingly stable. The R36A mutation caused about a six-fold increase in mini-F loss, the other mutations almost none, within measurement error. Assays of β-galactosidase showed that only the R36A mutant caused a significant rise in transcription, 1.8-fold (data not shown), a result confirmed by the 1.6-fold increase in SopA protein determined by immunoblot assay of cell extracts (Figure S1A). To determine whether higher *sopAB* expression accounted for the instability of the R36A mutant relative to the others, we assayed stability of mutant derivatives of the other mini-F, pDAG173, in which *sopAB* is transcribed constitutively from *pL-tetO* [27]. With Sop protein levels thus rendered uniform (though 3.4-fold higher than with pDAG218; Figure S1A), the *sopB* mutant derivatives of pDAG173 showed much the same degree of modest instability as their pDAG218 equivalents (Figure 1A); the increased loss rate of the R36K mutant is offset to some extent by the variability inherent in low loss rate estimates, such that we cannot assess its significance. The essential result is that the R36A, and possibly R36K, mutation results in slight destabilization of mini-F.

Attempting to increase the sensitivity of the stability assay, we reconfigured it by placing the *sopAB* operon under *plac* on an oligocopy (pSC101) vector, *in trans* to a mini-F *ΔsopAB* derivative. We considered that SopB protein radiating from the point of its production near the *sopAB* 3′ end might well be less plentiful at *sopC* owing to free diffusion and increased competition from non-specific DNA, resulting in formation of smaller, less effective SopB assemblies that amplify the handicap imposed by the mutant SopB defects. While wt *sopAB* still fully stabilized the mini-F (0.005% loss per generation) in this *trans* test, the mutations provoked much higher mini-F instability than when in *cis* (Figure 1B). The R36A mutation had the strongest effect, causing ∼1000-fold destabilization, about half that seen with no active partition, while the R36K and R42A mutations destabilized less, ∼360- and 240-fold respectively, and the R42K mutation not at all. Whether or not our rationale for applying the *trans* test is correct, by amplifying loss rates the test serves as an indicator of Sop system performance. All succeeding experiments were carried out using this *trans* configuration.

Alteration in SopB affinity for DNA, for SopA or for itself is unlikely to explain the partition phenotypes seen. We have shown that the mutant SopB proteins are unchanged with respect to specific and non-specific DNA binding, and exhibit affinity for SopA similar to that of wt SopB [12]. They also behave as wt SopB in destabilizing mini-F when in excess and in silencing transcription from a nearby promoter, two properties which depend on interaction of SopB dimers via their N-terminal domains (Figure S4). More importantly, the deficiency of each SopB in ATPase stimulation is closely correlated with the rate of mini-F loss (Figure 1B). Thus we can be confident that the restricted ability of the SopB R36A, R36K and R42A mutant proteins to stimulate SopA-mediated ATP hydrolysis is the major or exclusive cause of the defective partition seen. Because ATP hydrolysis is thought to be necessary for the segregation event that localizes plasmids in cell-halves, we next sought clues to the role of SopB-stimulated ATP hydrolysis by directly visualizing mini-F molecules.

### Mini-F clustering

**Figure 2:**
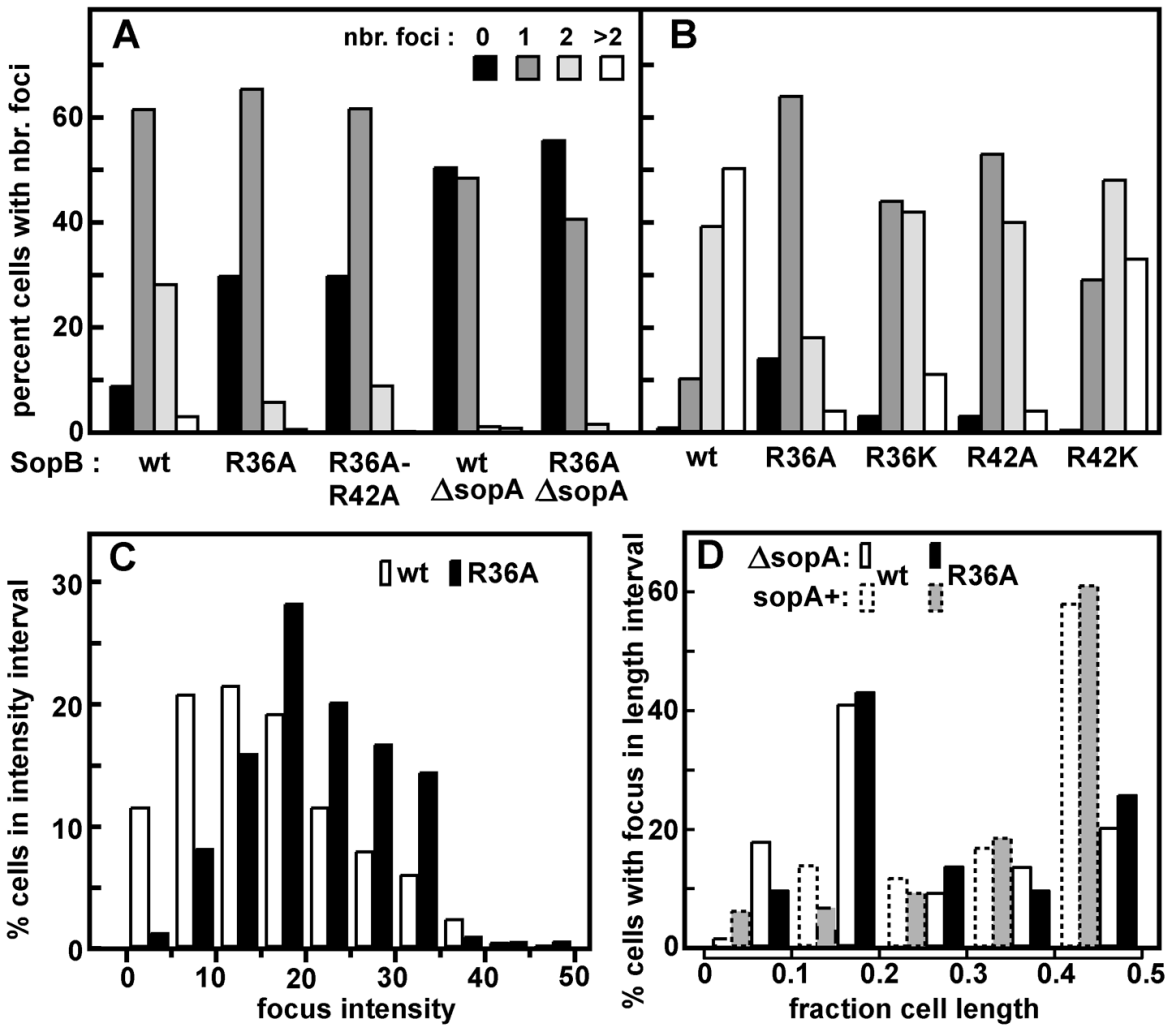
Effect of R36 and R42 mutations on mini-F clustering. Derivatives of strain DLT2583 (*para-tetR*::*gfp*) carrying the *tetO*-array mini-F, pDAG848, and producing SopA and mutant SopBs *in trans* were grown in MGC medium (A) or LB broth (B), and the mini-F plasmid foci were visualized as described in Materials and Methods and counted. Total cells scored were: A - 756 (wt), 626 (R36A) 415 (R36A, R42A); 344 (Δ*sopA*); 368 (Δ*sopA*, R36A); B - 2118 (wt), 3966 (R36A), 1874 (R36K), 1585 (R42A), 1736 (R42K). The wt and R36A focus number distributions in panel A were shown by the Student t-test to be different at a confidence interval of 95%. Sop protein concentrations relative to wt mini-F were measured by immuno-blot assay to be 1.9- and 2.2-fold (SopA) and 1.7 and 2.4-fold (SopB) higher in cells grown in MGC and LB respectively. C. Brightness of the foci in LB-grown cells, measured using Microbe Tracker. The distributions are different at the 95% confidence level by the Student t-test. Because focus intensity responds non-linearly to plasmid content (as seen elsewhere; e.g. [51]), foci in R36A cells were on average 1.3-fold brighter than in SopB-wt cells while consisting of 2.4 times as many molecules. D. Localization of pDAG848 without SopA. Cells were grown in MGC. The shortest pole-focus distances in single-focus cells were sorted into 1/10 cell-length classes. Dotted-line bars show distributions of single foci in the presence of SopA (Figure 5B). Distributions of focus brightness in Δ*sopA* cells containing wt and R36A SopB proteins were not significantly different at the 95% confidence level, by the same test as applied to the brightness data of Figure 2C (not shown).

To observe mini-F, we inserted an array of Tet repressor binding sites into a mini-F equivalent to that used in the *trans* stability tests, and introduced the resulting plasmid (pDAG848) into a TetR::Gfp-producing strain for visualization by fluorescence microscopy. The concentration of Sop proteins made from the pSC101-based plasmids is only about twice the wt mini-F level (Figure S1B; Table S1), owing to regulation of *sopAB* expression by Lac repressor in this MG1655 (*lacI^+^*)-based strain. Figure 2A shows that cells growing in MGC at 1.2 generations per hour, with an average of 3 copies of pDAG848 per cell, contained 1.2-fold more foci in the presence of wt SopB than of SopB-R36A. The difference was significant (see legend) but not strong, and was not increased by addition of the R42A mutation (Figure 2A). It was accentuated in cells growing in LB at 2.4 generations per hour with an average of 4.6 copies per cell [28], as shown together with foci/cell for the other *sopB* alleles in Figure 2B. An average of 3 foci per cell were seen in cells with wt SopB, 2.2 with the R42K mutant, 1.5 with R36K or R42A, and 1.2 with R36A. Consistent with this observation, R36A foci were on average brighter than wt foci (Figures 2C, S3A). Copy number per mini-F-carrying cell was not affected by the nature of the SopB protein (Table S2). Hence, whereas in the presence of wt SopB at least half the foci consist of a single plasmid molecule, the number of molecules per focus rises progressively as the ability of SopB to stimulate ATP hydrolysis declines, up to nearly four in the case of R36A. The frequency of cells with no focus also rises, consistent with destabilization by the R36A allele in LB-grown cells (Table S1). SopB-stimulated ATP hydrolysis apparently contributes to splitting mini-F clusters or preventing their formation.

To determine whether the R36/42 mutations might influence plasmid clustering independently of SopA we observed pDAG848 supplied with the SopB proteins only. Strong destabilization of mini-F by removal of SopA has been attributed to failure to resolve clusters [29], but inability to direct plasmid movement could also contribute [18], [22]. pDAG848 foci were indeed not seen in half the cells and seldom numbered more than one when present (Figure 2A). In addition, the preferred mid-cell positioning of single mini-F foci gave way to a more polar localization (Figures 2D, S3B). The same reduction in focus number and change in focus distribution occurred whether cells carried the wt or the R36A allele of *sopB*. Likewise, in the presence of SopA, fluorescent derivatives of wt and R36A SopBs both formed foci distributed over the long cell axis like those of labelled mini-F (Figure S3C). We thus saw no evidence for an effect of the R36/42 residues other than stimulation of ATP hydrolysis by SopA.

### SopA relocation

The elements that most effectively stimulate SopA to hydrolyse ATP, SopB and *sopC*, are also needed to activate the migration of SopA between nucleoid poles, implying that SopB mutants with significant deficiencies in ATPase stimulation, R36A, R36K and R42A, would reduce or eliminate SopA relocation. In order to examine dynamic behaviour of SopA in the presence of wild type and mutant SopB proteins, we introduced into the chromosome of both DLT1900 and DLT2583 a *sopA::xfp* fusion whose product fully stabilizes mini-F [18]; the construction restored *lacI* and *lacY* to DLT1900, putting *sopA::xfp* expression in this strain under LacI control and enabling the two strains to be used interchangeably. The DLT1900 derivative (DLT2687) was transformed with pYAS47 (pSC101-*plac*:*sopAB*) and pDAG209 (mini-F-Δ(*sopAB*) *sopC* ^+^), and the movement of SopA::Xfp was followed by fluorescence microscopy. As seen elsewhere [13], fluorescent SopA moved from end to end of the nucleoid 3–5 times per cell cycle provided *sopC* and wild type SopB were present (data not shown).

**Figure 3:**
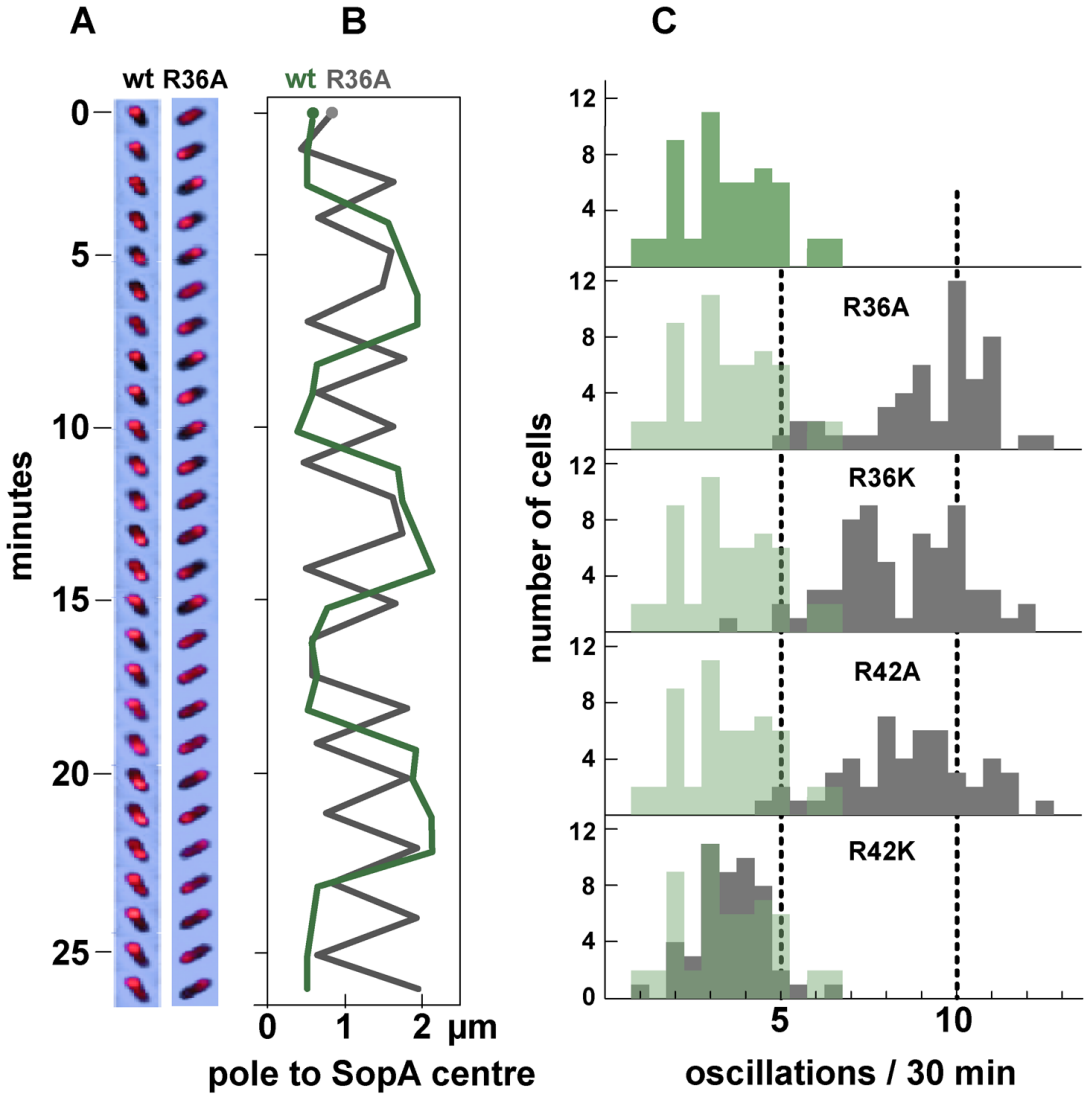
Effect of R36 and R42 mutations on SopA movement. A. Time-lapse image series of typical DLT2687 (*plac-sopA::xfp*)/pDAG848 (mini-F)) cells carrying pYAS47 (*sopA^+^B^+^*) or pYAS64 (*sopA^+^B* ^R36A^) grown in MGC-IPTG (0.1 µM) medium and applied to MGC-IPTG agarose slides. Images were acquired with an Olympus microscope at 70-second intervals for 29 minutes using 0.5 sec exposure. B. Kymograph of the SopA::Xfp foci in A. Each point represents the position of the brightest pixel in the image. C. Distribution of oscillation rates in cells producing the wt and the four mutant SopBs. Rate data are binned into 0.5 oscillation/30 min intervals. The levels of SopA::Xfp and SopA were, respectively, about 3-fold and 0.3-fold the wt mini-F levels (Figure S1C); repression of the plasmid-borne *plac* promoter is stronger than that of the chromosomal *plac* in the conditions used.

We then measured the frequency of SopA relocation after substituting the mutant R36 and R42 codons in *sopB*. The time-lapse sequences shown in Figure 3A illustrate the surprising result depicted in Figure 3C - a major increase in oscillation frequency for all mutants except R42K, which behaves as wt. The average period of migration to the far nucleoid pole and back was 3.2 minutes for the R36A mutant and 3.6 minutes for R36K and R42A, respectively 2.7- and 2.4-fold less than the return trip with wt SopB (8.6 minutes). The increase in SopA relocation frequency was only a little higher for the R36A mutant than for R36K and R42A, rather than proportional to ATPase stimulation deficiency as with mini-F in stability and clustering (Figures 1C, 2B); a factor other than reduced ATP hydrolysis may be rate-limiting at this high oscillation frequency. Total SopA levels in these experiments were 3–4 times higher than that in mini-F-carrying cells, but relocation frequencies were comparable at SopA concentrations ∼ten-fold higher still (Figure S2); the high frequencies are thus not a result of SopA excess.

**Figure 4:**
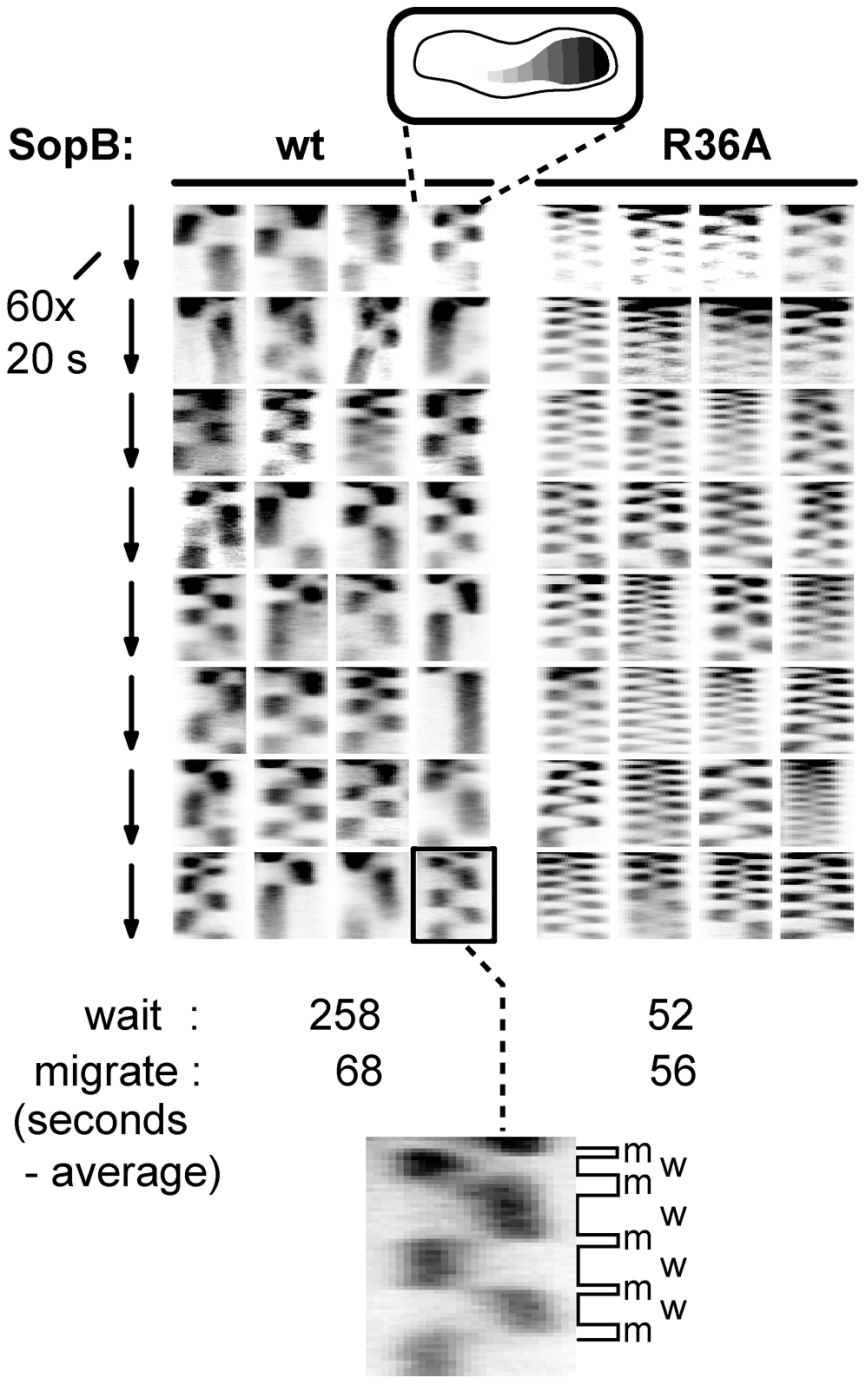
Effect of SopB-R36A on SopA relocation at high resolution. SopA::Xfp movement in DLT2740 (*plac-sopA::xfp*)/pDAG848 (mini-F)) cells carrying pYAS47 (*sopA^+^B^+^*) or pYAS64 (*sopA^+^B* ^R36A^) grown in MGC with 1 µM IPTG was monitored as in Figure 3 but with a Nikon microscope at 20 sec intervals for 20 min using 100 msec exposure. To facilitate comparison, kymograph widths representing cell length have been equalized and brightness has been adjusted. The kymograph at bottom has been expanded to show differentiation of SopA “wait” (w) and “migrate” (m) periods; wait is defined as an exposure in which >80% of the pixels are on one side of the mid-line, migrate as the interval between two waits.

The superimposed kymographs of Figure 3B suggested that the more rapid oscillation of SopA in the presence of mutant SopBs arose from virtual elimination of the normal pause between SopA's arrival at one end of the nucleoid and its setting out for the other. Accordingly, we repeated the experiment with shorter image-capture intervals. Because of the shorter viewing period the progressive decline of oscillation rate, owing to decelerating growth or other factors, has less effect, so that the average SopA return trip was shorter in this experiment, 1.8 minutes for R36A; nevertheless, the 3-fold increase relative to wild type was comparable to the 2.7-fold of 30-minute acquisition experiments, as shown in Figure 4. These kymographs confirm the initial indications that the difference in oscillation rates arises mainly from the short time that SopA spends at the nucleoid ends. The average speeds of SopA movement along the nucleoid were similar whether SopB was wt or mutant, whereas the average dwell-time decreased five-fold with the mutant SopB. Indeed the relative intensities of nucleoid-end signal in wt and mutant SopB cells suggest that in the latter, SopA arriving at the ends does not wait for following molecules before setting out again, although we have not ruled out the possibility that in this case much of the SopA is dispersed and relatively immobile. Western blotting showed similar levels of SopA::Xfp protein in both cases (Figure S1C); the difference is thus not due to blockage by piled up or aggregated protein. These observations imply that stimulation of ATP hydrolysis by SopB slows the process by which SopA reinitiates its migration but is not required for the migration itself.

Partition ATPase dynamics is generally thought to determine the trajectory and destination of segregating plasmid copies. Accordingly, we next investigated how the R36 and R42 mutations affect mini-F movement.

### Mini-F positioning and movement

**Figure 5:**
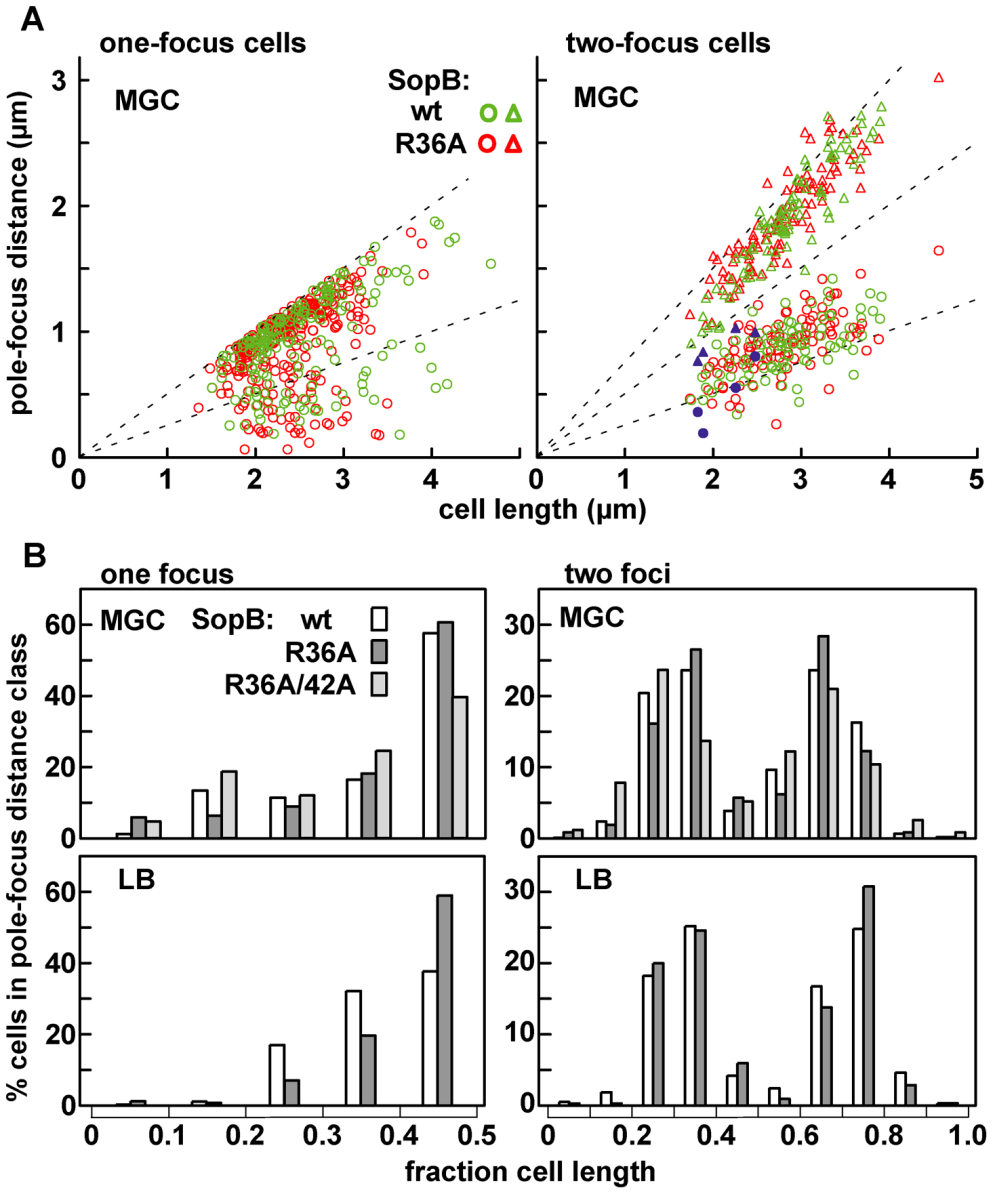
Limited effect of R36A mutation on mini-F positioning. The distance between the pole nearest a focus and the foci in MGC-grown cells of the one- and two-focus classes was measured. A: Pole-focus distances plotted against cell-length. The wt and R36A data-sets are superimposed. Blue symbols denote the four cases (out of 107, all R36A) in which both foci were unambiguously in the same cell-half. B: Pole-focus distances sorted into 1/10-cell intervals. In the right-hand panel distances to foci beyond the mid-point are measured from the same pole as the closer foci.

The average positioning of mini-F plasmids was little altered by substitution of SopB-R36A for wild type. Figure 5A shows the distances from pole to pDAG848 focus in the one- and two-focus classes of cells grown in MGC medium (Figure 2A); although R36A foci predominate at the margins of the distributions, the widening of the spread in R36A cells is slight. Figure 5B summarizes these data as plasmid focus position relative to cell length. Regardless of whether the cells were producing wt SopB or the most defective mutant, R36A, about 60% of single foci were within the central fifth of the cell and about 80% of double foci were grouped around the one-third and two-thirds positions. Similar positioning was seen in cells grown in LB medium (Figure 5B), despite the high degree of plasmid clustering observed in such cells (Figure 2B). Plasmid clusters appear to be as readily positioned as single molecules by the Sop partition apparatus. The most straightforward interpretation of this pattern is that the capacity of SopB to stimulate ATP hydrolysis does not strongly affect the average positions of segregation units once these have been defined by initial separation events. Nevertheless, abnormal positioning was evident in certain R36A cells whose two foci were clearly on the same side of mid-cell (blue symbols in Figure 5A); unless rectified before the next cell division this mis-positioning would result in partition failure.

**Figure 6:**
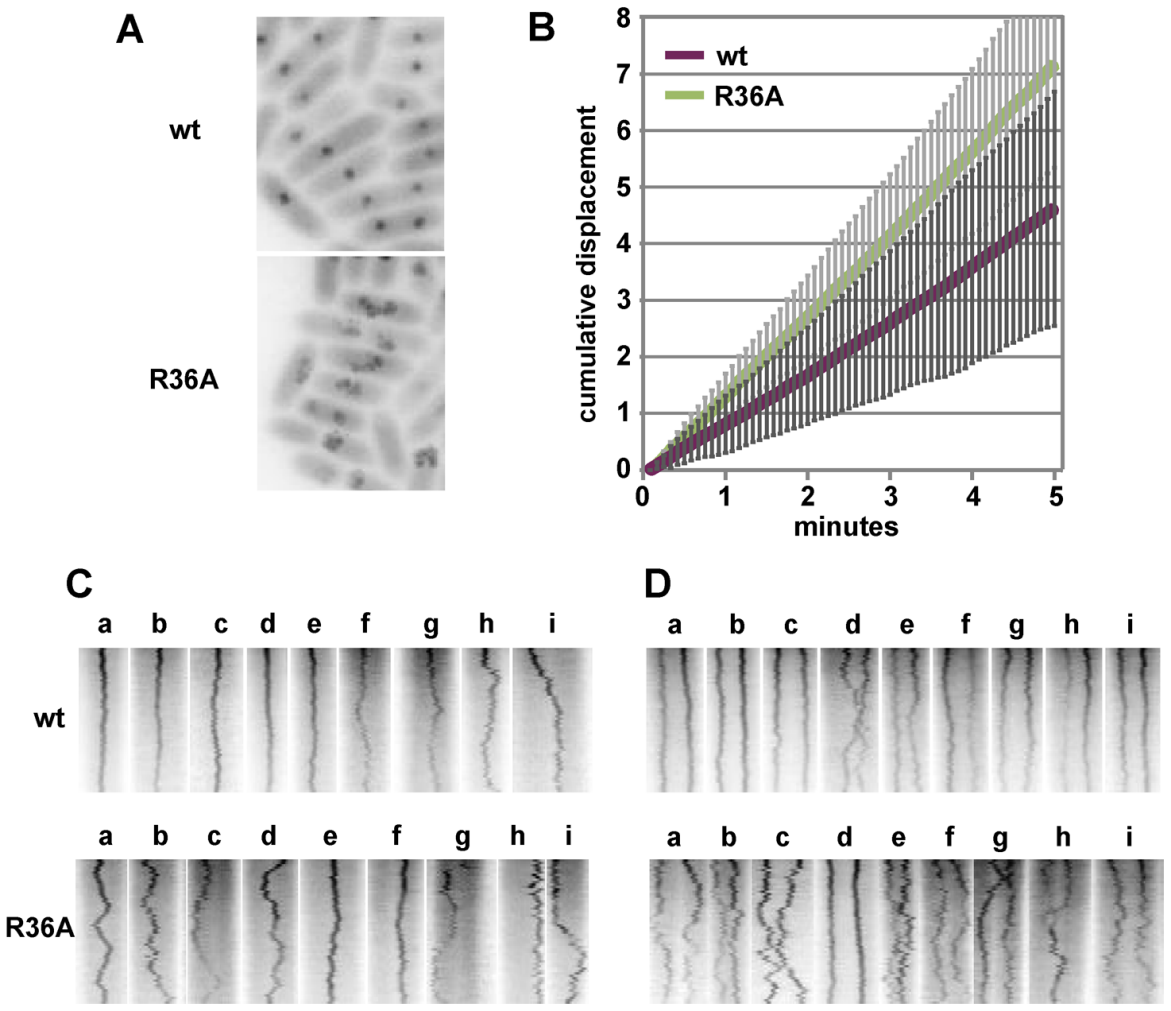
Effect of R36A mutation on mini-F movement. A. Cumulative displacement of mini-F, from images acquired every 5 secs for 5 mins, drawn using the Z project plug-in of Image J. B. Average cumulative displacement in 30 cells as illustrated in A; error bars are standard deviations. C. Illustrative individual kymographs of 1-focus cells; width corresponds to cell length (not equalized). D. As for C, 2-focus cells.

To examine mini-F movement, we observed pDAG848 foci in MGC-grown cells after labelling the plasmid by brief induction of *tetR*::*gfp*. Cumulative plasmid trajectories (Figure 6A) show mini-F movement to be less constrained in the presence of the R36A mutant than with wt SopB. By imaging pDAG848 in thirty single-focus cells at 5-second intervals over 5 minutes, we could estimate that on average it moves about 1.5 fold faster (from cumulative distance, Figure 6B). We discerned no consistent relationship between SopA and mini-F movements when these were monitored simultaneously but we could estimate from the oscillation data (above) that in the 5 min viewing time a SopA wave would pass on average once for wt SopB and three times for the mutant. The illustrative kymographs in rows 1 and 2 of Figure 6D reveal that compared to wt SopB, which generally maintains mini-F steady near the centre or quarter positions (row 1 - a–e; h and i show the two exceptions), the R36A mutant provoked a jumpy, exaggerated movement (row 2 - e, f are exceptions) and a frequent approach to the cell poles (c, g, h, i) not seen with wt. The frequencies of detectable direction reversal were similar, 13.6+/−5.5 (s.d.) for wt and 14.6+/−7.1 for R36A.

Kymographs of 23 two-focus cells (Figure 6E) showed the maintenance of focus position to be, again, fairly constant with wt SopB and erratic with the R36A mutant (d in each row shows exceptions). Nevertheless, the time-averaged locations of the foci corresponded to the quarter positions of the long cell axis, as determined earlier (Figure 5). Correlated deviations in the positions of both foci were infrequent - 3 for wt (e.g. h, i), 4 for R36A (e.g. e, f, i) - but instances of temporary occupation of a single cell-half by both foci were more common for the mutant SopB (7 - e.g. c, e, g, h) than for the wt (1 - d). In R36A mutant cells the two foci often approached each other closely, presumably a simple result of their wider ambit, and in one of the 50 cells examined in this experiment were seen to fuse (row 2 - h). The one close approach seen in 50 wt SopB cells (row 1 - d) did not result in fusion. This frequent and occasionally protracted positioning of both mini-F copies on one side of the eventual division septum in SopB-R36A cells, as seen in the four examples of Figure 5A, is consistent with the higher plasmid loss rate associated with this mutant (Figure 1B).

## Discussion

Our approach to defining the role of ATP hydrolysis differs from previous attempts, in that it targets the activator, SopB, rather than the instrument, SopA. The assumption that the behaviour of a mutant ATPase which binds but does not hydrolyse ATP is due to a defect in hydrolysis *per se* can be misleading; inability to hydrolyze ATP may be a secondary consequence of the mutation or be relevant only at a later step in the pathway. For example, the partition disabilities initially attributed to defective ATP hydrolysis by the K122Q mutant of P1 ParA [30] and the K120R mutant of SopA [7] are now known to be caused by impairment of conformational transitions, seen by changes in tryptophan fluorescence [23] and in CD spectrum (helicity) [7] respectively; these conformational shifts allow the wild type proteins to bind to DNA [23] and to polymerize (our unpublished data), activities which require ATP binding but not hydrolysis. Other observations of reduced ParA dynamics associated with mutations that diminish ATPase activity [6], [13], [15], [16] might require reinterpretation.

We find that when hydrolysis itself is unambiguously targeted, the consequences for plasmid stability are surprisingly modest. Even the SopB-36A mutant, which generates only 5% of normal ATPase activity, allows relocation of SopA along the nucleoid at normal speed, movement of plasmids and a high level of plasmid stability. Indeed we could obtain accurate estimates of partition deficiency only by distancing the *sopAB* operon from the mini-F test plasmid. Our initial presumption was that reduction of SopB concentration near *sopC* aggravates the partition deficiencies of the SopB mutants, but other explanations are possible; for example the ten-fold higher average level of Sop proteins in this experiment (Figure S1A) might alter SopA adherence to the nucleoid, as seen *in vitro* [31]. The possibility that the weak partition phenotype results from a smaller deficit in ATPase stimulation *in vivo* than that measured *in vitro* must be put aside provisionally; the lack of a specific *in vivo* ATPase assay prevents its being tested. The further possibility that the R36 and R42 mutations also affect SopA activity in a way that compensates for loss of ATPase stimulation is not formally excluded but is rendered unlikely by the strong correlation of increased plasmid instability and clustering with *in vitro* ATPase reduction (Figures 1B, 2). Such instability as there is appears to stem mostly from plasmid clustering or failure to split clusters (Figure 2).

This finding does not sit comfortably with current views of the partition mechanism. The primordial model of Leonard et al [20] and those evolved from it [15], [16], [22], [23], [32], [33] posit hydrolysis of ATP as the primary activator of ParA relocation and hence of plasmid segregation. Binding of ATP induces SopA, like other ParA proteins, to undergo a conformational change that enables it to associate with non-specific DNA [21]–[23] and thereby to coat the nucleoid [13], [14], [18], [34]. It also promotes interaction of SopA with SopB [12]. According to these models, the mini-F partition complex thus encounters SopA-ATP primed by contact with the nucleoid to hydrolyse ATP, and prompts it to do so. After hydrolysis SopA can no longer bind to DNA and departs for the cytoplasm where it reacquires ATP and binds to DNA further along the nucleoid, setting up a wave-like relocation between the nucleoid ends. By acting as a mobile attractor, the SopA wave creates and maintains distance between plasmid copies [35]–[38]. All type I plasmid partition systems studied conform to this general description, regardless of differences in detail.

Thus the main role of ATP hydrolysis is currently thought to be dislodgement of SopA from the nucleoid. But our results (Figures 3, 4) show that SopA relocation, which requires detachment from the nucleoid, is not impeded by severely weakened hydrolysis. The bulk of SopB-stimulated ATP hydrolysis thus appears to be unneeded for SopA detachment from the nucleoid and relocation. On the other hand, basal hydrolysis stimulated two-fold by DNA or residual SopB-mediated stimulation (5% for SopB-R36A) may well suffice to activate or maintain a largely auto-propagating mode of SopA relocation. The observation that pB171 ParA::Gfp continues to retract polewards after disconnection from ParB [16] is in line with this possibility. But if so, a role other than SopA detachment from the nucleoid must be sought for most of the ATP hydrolysis that SopB stimulates. Furthermore, other mechanisms of SopA detachment must be considered. One possibility is competition by the SopB molecules massed in the extended partition complex for the nucleoid surface occupied in meta-stable fashion by SopA-ATP, which could be examined using the approach of Havey *et al.* [39] for observing behaviour of dynamic complexes of the P1 Par system components *in vitro*. An alternative is suggested by the recent finding that in an *in vitro* analogue of the Sop system SopB enhances dissociation of SopA-ATP from a DNA surface [31]. The authors suggested that SopB induces a conformational change in SopA that weakens its affinity for DNA and potentiates subsequent ATP hydrolysis. Specific removal of hydrolysis from this process would generate SopA-ATP unable to immediately rebind the nucleoid but primed to relocate along it. Indeed an even stronger effect of SopB revealed in this study was the inhibition of SopA-ATP association with DNA, implying that once removed from the nucleoid SopA-ATP would be unable to reassociate with it until outside the extended partition complex zone.

**Figure 7:**
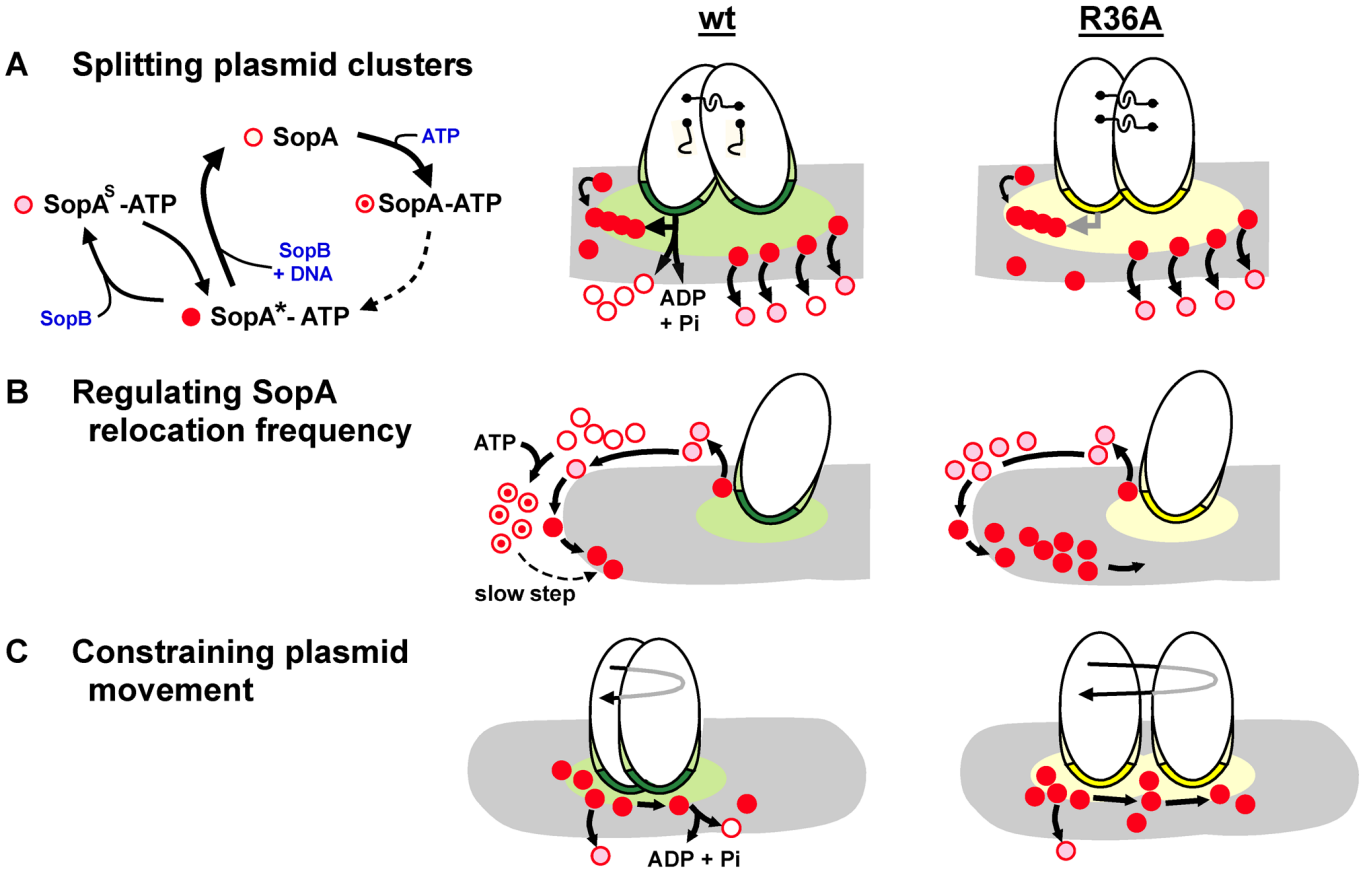
Steps in partition at which SopB stimulates SopA to hydrolyse ATP. Mini-F plasmids are shown oval with SopB bound to *sopC* in the partition complex as dark green (wt) or yellow (R36A) and SopB spread *in cis* or onto the nucleoid (grey) in the corresponding lighter shade. No distinction is made here between direct binding of SopB to the nucleoid and binding via SopA tethers; the latter option is favoured by recent observations of partition complex movement as a function of SopA-ATP disassociation from a DNA carpet [31]. Distinct functional forms of SopA are shown in the key as: SopA*-ATP, able to bind to non-specific DNA (corresponding to ParA*_2_-ATP_2_; [23]); SopA^S^-ATP, modified by SopB to be unable to bind non-specific DNA [31]; SopA, without ATP following hydrolysis; SopA-ATP, associated with ATP but not having undergone transition to the DNA-binding form [23]. A. Model of SopB-stimulated splitting of clusters. SopB spreads from clustered partition complexes, creating a mat which attracts SopA-ATP and shelters it from the nucleoid to allow SopA polymerization [21]. For clarity only one polymer (chain of red dots) and its interactions is shown, at the left of the SopB mat. Polymer contact (horizontal arrow) with SopB-*sopC* initiates a hydrolysis-driven transfer of each partition complex to successive polymer subunits which generates the force to break inter-plasmid links (shown as hooks). Hydrolysis stimulation by SopB-R36A is too low to drive this process efficiently. Following splitting, the SopB mat shrinks, attenuating polymerization and this mode of plasmid separation. The separation mode that positions plasmid molecules (e.g. diffusion-ratchet; [20], [23]; shown at right of SopB mats) is mostly independent of ATP hydrolysis [31] and is activated by both wt and R36A SopB complexes. B. Model of SopA priming for relocation. Relatively slow conformational transitions [23] must raise the level of SopA-ATP competent to start relocation from the pole. Most of the SopA-ATP dislodged by the R36A partition complex does not hydrolyse its ATP, does not need the transition, and so reverses direction without delay. C. Model of restricted F plasmid movement. The extended partition complex of a segregated plasmid encounters nucleoid-bound or relocating SopA molecules at random and moves as dictated by local SopA dynamics. Wt SopB complexes stimulate ATP hydrolysis, disrupting SopA-SopB contact [35] and curtailing movement, whereas R36A complexes do not, leading to a wider ambit (longer reverse arrow), accounting for the exaggerated position adjustments seen in Figure 6.

Figure 7 illustrates the three partition processes in which we propose ATP hydrolysis to play a direct role. Hydrolysis-independent dissociation from DNA, as discussed above, would help account for one of them, the high frequency of SopA::Xfp oscillation seen in response to the SopB R-finger mutants (Figures 3, 4). In the presence of wt SopB, SopA waves were interrupted by a waiting period at the nucleoid poles (as also seen by Hatano *et al.* [13]), whereas with the R36A mutant this period was very brief, perhaps non-existent at the level of the individual SopA molecule. The difference implies that SopA molecules can arrive at the nucleoid tip in distinct forms that determine their ability to reverse direction. SopA arriving in response to the R36A mutant is already competent to relocate and does so immediately, whereas SopA stimulated by wild type SopB requires adaptation before setting out again, as illustrated in Figure 7B. This conditioning could consist of a time-consuming conformational change in the ATP-bound form of SopA, such as that culminating in ability of P1 ParA to bind DNA [23]. A similar case of apparent reconditioning at nucleoid ends was reported by Autret & Errington [40], who observed that the MinD division topology factor was implicated in relocation of the SopA homologue, Soj, from nucleoid ends in *Bacillus subtilis*. In a prior study [41] these authors had isolated a mutant of the cognate SopB homologue, Spo0J, (*spo0J19*) that increased the frequency, but not the speed, of Soj relocation. Modelling of Soj dynamics led Doubrovinski & Howard [36] to propose that Spo0J19 might increase relocation frequency by expelling Soj from the nucleoid in a form that rebinds the nucleoid ends without involving MinD, clearly analogous to bypassing the SopA conditioning that we suggest is a normal prelude to relocation. Our observations suggest that ATP hydrolysis might function as a means of restricting the rate of SopA relocation. Such regulation could be needed to maintain stable positioning of the plasmid.

The effects of the mutant SopBs on mini-F positioning are intuitively easier to relate to plasmid stability. As well as driving segregation of mini-F copies, SopA restricts their subsequent movement [35]. This restriction broke down when the partition complexes contained SopB-R36A instead of wild type. Most of the traces shown in Figure 6 are presumably of positioned mini-F molecules not in the first phase of active segregation. ATPase stimulation is thus necessary for positional stability. If ATP hydrolysis normally repositions plasmids by removing SopA from the nucleoid, we would expect R36A partition complexes to move more sluggishly than wild type, not faster as seen. It is possible that the duration of contact between SopA-ATP and the partition complex is the key determinant of plasmid movement in this context, such that the longer contact expected from weak hydrolysis stimulation by an R36A partition complex might result in the more prolonged pulling of the plasmid by retracting SopA, accounting for the greater displacements seen (Figure 6; depicted in Figure 7C). Regardless of mechanism, the wider ambit of mini-F carrying SopB-R36A could lead to instability by increasing the chance of all plasmid copies finding themselves in the same cell half or in clusters (Figure 6D) at division. There is reason to question, however, whether temporary mis-positioning and cluster formation are the major causes of instability - fluorescent foci of various wild type plasmids have been seen to fuse [16], [42], [43] and fusion/splitting may be part of normal positioning [44]. The greater threat to stability might be failure to break clusters formed immediately after plasmid replication.

The inter-plasmid links that form clusters are not based on pairing of partition complexes [45], but active partition breaks them [46]. We suggest that segregation of recent replicas requires ATP hydrolysis to play a different role from the triggering of a conformational switch that releases SopA from the nucleoid at later stages of positioning [31], namely provision of energy for the initial segregation event. We proposed previously that following replication SopB spreads from the partition complex onto nucleoid DNA, creating a DNA-free zone that permits SopA to polymerize and thus initiate partition [21]. The recent observation that in an *in vitro* reconstituted system SopA-ATP does not form filaments on a DNA carpet is consistent with this proposal [31]. Our evidence that clustering is accentuated in proportion to the deficit in ATPase stimulation (Figure 2) encourages a speculation on the role of SopA polymerization - the forced separation of clustered plasmids powered by hydrolysis of ATP (portrayed in Figure 7A), as an initial step that enables partition complexes to then be drawn apart by the retreating SopA wave. While we have no direct evidence for this proposal, it is compatible with the two-phase segregation of F plasmids observed by Gordon *et al.* [47], in which a very rapid initial separation was followed by gradual progress to the destination. Biochemical and microscopical study focused on this aspect of SopA/B behaviour is now needed to test the proposal

In summary, our results reveal that SopB-mediated stimulation of SopA-catalyzed ATP hydrolysis serves mainly to split F plasmid clusters following replication and to prevent them forming later. SopA relocation and plasmid movement might depend on basal ATP hydrolysis but need little or no further stimulation by SopB. SopB-stimulated hydrolysis appears rather to regulate SopA relocation frequency. The contribution of basal hydrolysis could be approached using SopA mutated at residues directly involved in catalysis whose minimal conformational effects can be demonstrated. This altered perspective should be taken into account in attempts to determine how the partition of other replicons works.

## Materials and Methods

### Bacterial strains and growth conditions

The strains are derivatives of *E.coli* K12 and are listed in Table 1. DH10B was used as a transformation recipient for routine plasmid construction. The base strains for mini-F loss rate determinations were MC1061 and DH10B. For microscopic observation, the following mutations were introduced by recombineering, or by P1 transduction from the recombineerants: Δ(*araFGH*) and Ω*pcp*18::*araE*533 into MG1655 as in the construction of DLT1900 [9] to moderate arabinose induction, Ω(*para*-*tetR*::*egfp*) to follow movement of mini-F (pDAG848; see below), and Δ*lacZ*Ω(*plac-sopA*::*xfp*) to monitor SopA relocation; details of the two latter constructions are given in Supplementary Information (Text S1). Cultures were grown at 37°C with aeration in Luria-Bertani (LB) broth, or in M9 salts-thiamine (1 µg/ml) supplemented with 0.4% glycerol and either 0.2% Casamino acids (MGC medium) or 20 µg/ml leucine, as noted in the text. When needed for selecting plasmid maintenance, antibiotics (Sigma) were added (µg/ml): kanamycin (30), spectinomycin (30), chloramphenicol (10).

**Table 1:** Strains and plasmids.

Strain	Genotype/relevant properties	Source/reference
DH10B	F^−^, *mcrA*, Δ(*mrr-hsdRMS-mcrBC*), Φ80*dlacZ*ΔM15, Δ*lacX74*, *endA1*, *recA1*, *deoR*, Δ(*ara,leu*)*7697*, *araD139, galU, galK, nupG, rpsL*	[55]
MC1061	*araD139, Δ(ara-leu)7679, ΔlacX74, galU, galK, rpsL, thi, sdR2, mcrB*	[56]
MG1655	F^−^, λ^−^	[57]
DLT1900	MC1061 Δ*araFGH*, Ω*pcp18*::*araE533*	[9]
DLT2202	MG1655 Δ*araFGH*, Ω*pcp18*::*araE533*	This work
DLT2583	DLT2202 Δ(*araBAD'*)-Ω(*para-tetR::gfp*)	This work
DLT2687	DLT1900 Δ*lacZ* Ω(*plac-sopA::xfp*)	This work
DLT2740	DLT2583 Δ*lacZ* Ω(*plac-sopA::xfp*)	This work
DLT2721	DLT2687 pAM238	This work
DLT2722-6	DLT2687 pDAG209, pYAS47, -64, -65, -66, -67	This work
DLT2891	DLT2583 pDAG848, pAM238	This work
DLT2734-8	DLT2583 pDAG848, pYAS47, -64, -65, -66, -67	This work
DLT2867	DLT2740 pDAG848, pAM238	This work
DLT2741-2	DLT2740 pDAG848, pYAS47, -64	This work
Plasmid	Relevant characteristics	
pAM238	*lacOPZ'*Ωmcs in *Eco*RI-*Hind*III interval of pGB2, *aadA* ^+^	[58]
pDAG114	*repFIA*, *ccdB* ^−^, *resD* ^+^, *rsfF* ^+^, *cat*	[59]
pDAG173	pDAG114 with *sopAB* control replaced by *pLtetO*	[49]
pDAG209	pDAG114 Δ*sopAB*	[59]
pDAG218	pDAG114 with *lacZ* inserted between *sopB* and *sopC*	This work
pDAG848	pDAG209, *tetO* array	This work
pDAG781	pDAG218 *sopB*-R36A	This work
pDAG782	pDAG218 *sopB*-R36K	This work
pDAG783	pDAG218 *sopB*-R42A	This work
pDAG784	pDAG218 *sopB*-R42K	This work
pYAS29	pDAG173 *sopB*-R42A	This work
pYAS37	pDAG173 *sopB*-R42K	This work
pYAS43	pDAG173 *sopB*-R36A	This work
pYAS45	pDAG173 *sopB*-R36K	This work
pYAS47	pAM238 *plac-sopAB*	This work
pYAS64	pYAS47 *sopB*-R42A	This work
pYAS65	pYAS47 *sopB*-R42K	This work
pYAS66	pYAS47 *sopB*-R36A	This work
pYAS67	pYAS47 *sopB*-R36K	This work
pYAS70	pYAS47 Δ(*sopA*_115–358_)	This work
pYAS71	pYAS64 Δ(*sopA*_115–358_)	This work
pYAS81	pYAS47 *sopB*-R36A, R42A	This work
pJYB259	pYAS47 *sopB*::*mVenus*	This work
pJYB260	pYAS64 *sopB*::*mVenus*	This work

### Plasmids

Plasmids are listed in Table 1. Construction outlines are given here, with details available on request. pDAG218: *lacZ* with its Shine-Dalgarno sequence was incorporated in the *sopAB* operon by insertion into the 75 bp *Acc*I-*Apa*I interval between *sopB* and *sopC* in pDAG114. pDAG848: deletion of *sopAB* from pDAG114 and insertion 195 bp downstream from *sopC* of 2.36 kb of the *tetO* array of pFX240. pYAS47: insertion of a PCR fragment containing *sopAB* with its Shine-Dalgarno sequence downstream from the *lac* promoter in pAM238. *sopB* alleles encoding the R36A-, R36K-, R42A- and R42K mutant SopBs were introduced into pDAG173, pDAG218 and pYAS47 by restriction fragment replacement to yield the derivatives listed in Table 1. pYAS70,-71: in-phase deletion of *sopA* codons 115–358 by cleavage with *Bst*XI and *Sap*I, Klenow end-filling and ligation. pYAS81: introduction of R36A (*sopB* nt106-7 CG>GC) to pYAS66 by quick-change mutagenesis. pJYB259,-260: insertion of a PCR fragment containing a fusion of *sopB* to mVenus [48], a derivative of Venus rendered monomeric by mutation A207K (this laboratory).

### Mini-F stability

Cells grown with selection for mini-F plasmids were diluted into antibiotic-free medium and the resulting cultures sampled at intervals for assay of the plasmid-free fraction as follows. pDAG173 and derivatives, and pDAG209: cells were spread on LB agar and the resulting colonies tested by replica plating to selective medium [49] or by the three-layer method described [50]. pDAG218 and derivatives: cells were spread on MGC agar containing X-gal (40 µg/ml) and white and blue colonies scored after incubation.

### Fluorescence microscopy

Cells of strains expressing *sopA*::*xfp* from the chromosomal *lac* promoter, DLT2687 and DLT2740, or *tetR::gfp* from the tet promoter, DLT2583 and DLT2740, were transformed with the mini-Fs pDAG848 or pDAG209 and pSC101-based vectors expressing *sopAB*. Transformants grown overnight in MGC medium were diluted 1/400 into MGC (except as noted), supplemented in the case of DLT2740 derivatives with IPTG at 0–10 µM (see Results), and grown at 30°C to OD_600_ ∼0.25, then sampled for observation; for visualization of pDAG848, *tetO::gfp* was induced by addition of arabinose to 0.05% 30 min beforehand. Samples were processed for microscopy as described [18]. Slides were maintained at 30°C in a thermostated chamber and examined by phase contrast and fluorescence microscopy using inverted microscopes: (i) Olympus IX81, images captured with a Coolsnap 2 (Roper) camera and processed using Metamorph software; (ii) Nikon Ti-E, acquisition with a EMCCD (Hamamatsu) camera (gain 144), and processing with NIS- element software. Metamorph software was used to generate kymographs, and to measure absolute distances (x,y) of mini-F displacement between images with the “Track Object” tool.

## Supporting Information

Figure S1 Immunoblot assay of SopA concentration *in vivo*. Cells from cultures corresponding to those used to examine mini-F stability (A: left panel – DLT1900, M9-glycerol; centre – DH10B, MGC; right – DLT1900, M9-glycerol), mini-F distribution and movement (B: left – DLT2853, MGC; right – DLT2853, LB) and SopA(::Xfp) relocation (C: left – DLT2687, MGC-IPTG 0.1 µM; right – DLT2740, MGC-IPTG 0.1 µM) were centrifuged, washed with 50 mM NaCl-1 mM EDTA-10 mM Tris pH 7.4, resuspended in SDS-sample buffer [52] at 3.3 OD_600_ units/ml and incubated at 95°C for 5 min; lysates were vortexed for 10 sec, chilled on ice, centrifuged and analyzed immediately by PAGE or stored at −20°C for further use. The volumes shown (µL loaded) were brought to 12 µl with an equivalent extract of background-strain cells, then loaded on a 4–12% bis-Tris gradient polyacrylamide gel (NuPage; Novex) in MOPS-SDS buffer and subjected to electrophoresis at 200 V for 50 min. Dilutions of purified SopA protein in MC1061 extract were treated in the same way to provide a standard curve (D). Separated proteins were electro-transferred to nitrocellulose membranes (IBlot gel, InVitrogen) and immuno-detected essentially as described [53], using anti-sera (Eurogentec) raised against purified SopA and SopB proteins and affinity-purified using membrane-immobilized samples of these proteins. wt – pDAG114, wt *lacZ* – pDAG218, R36A – pDAG781, R36K – pDAG782, pL*tet* AB^R36A^ – pYAS43, p*lac* AB – pYAS47, p*lac* AB^R36A^ – pYAS64. Negative controls shown are: Δ – pDAG415 (A, left), cfe – cell-free extract (B, right), p*lac* – pAM238 (C, left). Proteolysis products (SopA::xfp''s; ≤9% total) are included in the SopA::Xfp concentrations. Values used for concentration calculations were within the range so labelled.
(TIF)

Figure S2 Effect of excess *sopA* and *sopA*::*xfp* expression on relocation rate. The top panel is from Figure 3 panel 2, the bottom from an identical experiment with IPTG at 10 µM which raised the concentration of SopA from 0.3 to 7 mini-F units and of SopA::Xfp from 3.3 to 47 units. Arrows show average end-to-end relocations per 30 min.
(TIF)

Figure S3 Representative images of cells with labelled mini-F and SopB protein. A. DLT2583 cells carrying mini-F (pDAG848) labelled with TetR::Gfp and producing SopA and wt and R36A mutant SopBs *in trans*, used to determine mini-F foci per cell (Figure 2A) and focus brightness (Figure 2C). Note that in general the R36A foci are less numerous and brighter. B. DLT2583 cells carrying mini-F (pDAG848) labelled with TetR::Gfp and producing wt and R36A mutant SopBs *in trans*, used to determine mini-F foci per cell (Figure 2A) and distribution (Figure 2D) in the absence of SopA. Regardless of the *sopB* allele, foci are generally single, polar and brighter than with SopA present. C. DLT2202 cells carrying mini-F (pDAG209) and producing wt and R36A SopB::mVenus from pJYB259 and 260 respectively.
(TIF)

Figure S4 Auto-interaction of mutant SopB proteins. To test whether the *sopB* R36A and R42A alleles might affect partition by altering the ability of SopB protein to interact with itself (“oligomerize”) we incorporated the two mutants in a separate study aimed at mapping domains of interaction within the SopB N-terminus. The results for these and nearby mutations are presented here. Two indicators of *in vivo* SopB-SopB interaction were used: silencing of a *sopC*-proximal promoter, which results from spreading of the specific SopB-*sopC* complex along adjacent DNA by recruitment of further SopB dimers; and destabilization of a *ΔresD* mini-F (“IncG incompatibility”), which appears to depend on induction of mini-F pairing/clustering by excess SopB [9]. Silencing of the *p_aadA_*::*lacZ* fusion in strain DLT2684 (DLT2067 [9] with *gen* substituted for *cat*) was measured by introducing the *p_araBAD_*::*sopB* plasmid pDAG170 [21] and its *sopB* mutant derivatives, and measuring β-galactosidase specific activity following exponential growth of the resulting strains for about four generations in LB broth supplemented with 0.4% glucose, 20 µg/ml chloramphenicol and 10^−4^ M arabinose. To confirm the presence of the mutant SopBs at concentrations similar to that of the wt protein, samples of the same cultures were analyzed by electrophoresis in bis-tris denaturing gels (NuPAGE; Invitrogen), where they appear as a light but distinct ∼40 kD band (not shown). Mini-F destabilization was measured by introducing the same plasmids into DLT1900 (DLT2067 without *p_aadA_*::*lacZ*) carrying the Δ*resD* mini-F, pZC209 (Spc^R^; [28]), growing the resulting strains for about seven generations as above, spreading samples on non-selective LB agar and replica-plating the colonies on LB agar supplemented with 30 µg/ml spectinomycin. The top bar shows the known functional anatomy of SopB. The relevant N-terminal region is magnified to show the double, triple and R-finger mutants analyzed. Silencing data from 4–6 separate determinations for each mutant is shown as the ratio of silenced to unsilenced (Δ*sopB*) expression level (second bar; average ∼250 Miller units). Mini-F destabilization (IncG) data from 3 determinations (except the GVM mutant - one only) is shown as the logarithm of the fraction of population still carrying mini-F after ∼7 generations. Error bars are standard deviations. Both tests of SopB autointeraction (oligomerization - [54]) yield the same profile. The R36A and R42A mutants behave as the wild type. Slight loss of silencing and destabilization capacities by the GN>AA mutant and strong loss by GRD>AAA suggest that these residues mark one border of the SopB-SopB interaction domain.
(TIF)

Table S1 Effect of the sopB-R36A mutation *in trans* on mini-F (Δ(*sopAB*), *sopC* ^+^) stability. LR - % mini-F lost per generation, average of two or three determinations; SF - stabilization factor (LR_Δ*sopB*_/LR*_sopB_*_+/R36A_); DsF - destabilization factor (LR*_sopB_*_R36A_/LR*_sopB_*_+_); U-mF – concentration of SopA relative to that in cells carrying wt mini-F (pDAG114), determined independently for each of the strain-growth medium combinations. Corresponding SopA concentrations are shown, as the average values obtained for cells expressing the wt and R36A *sopB* alleles. * denotes data shown in Figures S1 A and B, other data are from parallel determinations not shown. Because *sopAB* expression is subject to LacI control in DLT2583 SopA concentrations are lower than in the Δ(*lacIZYA*) strain, DLT1900; this contributes to the low stabilization factors seen with wt SopB and consequently the lower destabilization factors seen with SopB^R36A^, relative to those obtained with DLT1900.
(DOC)

Table S2 Copy number of mini-F pDAG209 (relative to pAM238) provided with wt and mutant SopB proteins. DNAs in mini-preps from cells growing exponentially or in stationary phase in LB at 30°C were linearized by digestion with *Pci*I and the fragments quantified by gel-electrophoretic resolution, ethidium bromide staining and fluorescence measurement. Values are averages of two determinations. Fluorescence intensity measurements were corrected for the difference in length of the mini-F (7.6 kb) and the SopB-producing derivatives of pAM138 (6.4 kb), to enable calculation of copy number ratios.
(DOC)

Text S1 Construction of strains carrying Ω (*para-tetR::egfp*) and ΔlacZΩ (*plac-sopA::xfp*).
(DOC)
