# Supplementary figures and images for: Defining the Role of ATP Hydrolysis in Mitotic Segregation of Bacterial Plasmids
Source: PLoS Genet. 2013 Dec 19;9(12):e1003956. doi: 10.1371/journal.pgen.1003956 (PMC3868542; doi:10.1371/journal.pgen.1003956)

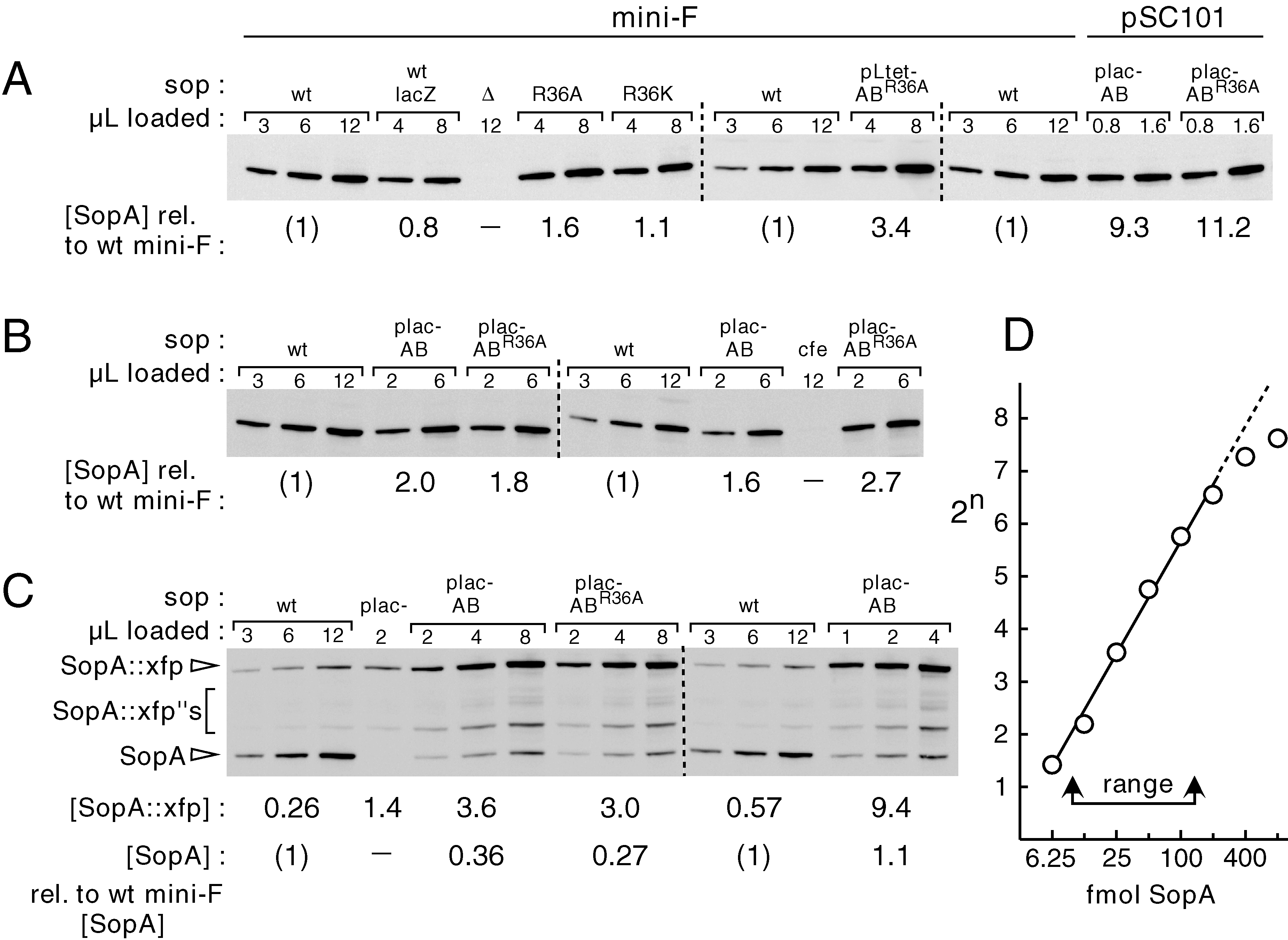

Supplement: Figure S1 — Immunoblot assay of SopA concentration in vivo. Cells from cultures corresponding to those used to examine mini-F stability (A: left panel – DLT1900, M9-glycerol; centre – DH10B, MGC; right – DLT1900, M9-glycerol), mini-F distribution and movement (B: left – DLT2853, MGC; right – DLT2853, LB) and SopA(::Xfp) relocation (C: left – DLT2687, MGC-IPTG 0.1 µM; right – DLT2740, MGC-IPTG 0.1 µM) were centrifuged, washed with 50 mM NaCl-1 mM EDTA-10 mM Tris pH 7.4, resuspended in SDS-sample buffer [52] at 3.3 OD600 units/ml and incubated at 95°C for 5 min; lysates were vortexed for 10 sec, chilled on ice, centrifuged and analyzed immediately by PAGE or stored at −20°C for further use. The volumes shown (µL loaded) were brought to 12 µl with an equivalent extract of background-strain cells, then loaded on a 4–12% bis-Tris gradient polyacrylamide gel (NuPage; Novex) in MOPS-SDS buffer and subjected to electrophoresis at 200 V for 50 min. Dilutions of purified SopA protein in MC1061 extract were treated in the same way to provide a standard curve (D). Separated proteins were electro-transferred to nitrocellulose membranes (IBlot gel, InVitrogen) and immuno-detected essentially as described [53], using anti-sera (Eurogentec) raised against purified SopA and SopB proteins and affinity-purified using membrane-immobilized samples of these proteins. wt – pDAG114, wt lacZ – pDAG218, R36A – pDAG781, R36K – pDAG782, pLtet ABR36A – pYAS43, plac AB – pYAS47, plac ABR36A – pYAS64. Negative controls shown are: Δ – pDAG415 (A, left), cfe – cell-free extract (B, right), plac – pAM238 (C, left). Proteolysis products (SopA::xfp''s; ≤9% total) are included in the SopA::Xfp concentrations. Values used for concentration calculations were within the range so labelled. (TIF) [file pgen.1003956.s001.tif]

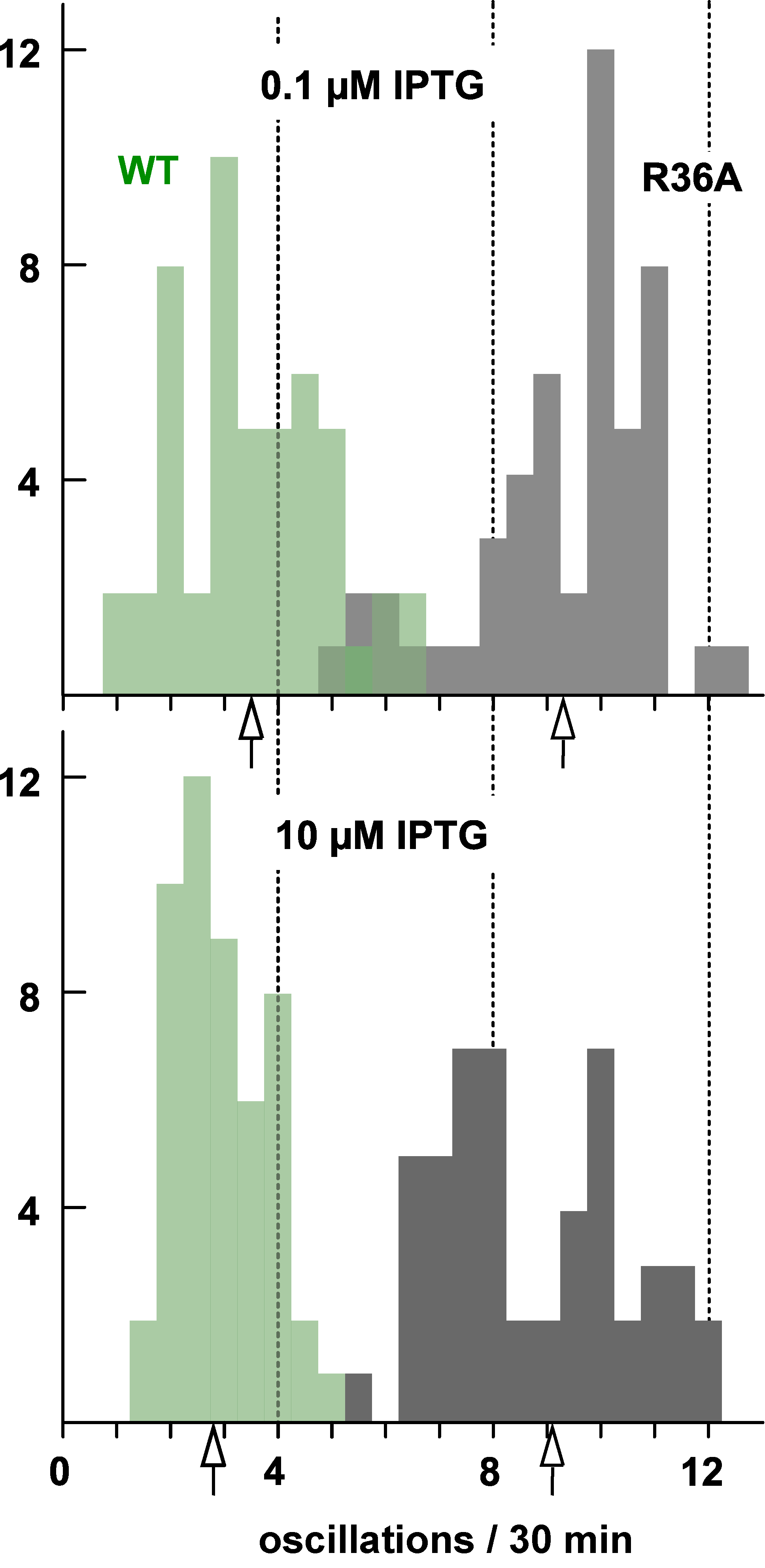

Supplement: Figure S2 — Effect of excess sopA and sopA::xfp expression on relocation rate. The top panel is from Figure 3 panel 2, the bottom from an identical experiment with IPTG at 10 µM which raised the concentration of SopA from 0.3 to 7 mini-F units and of SopA::Xfp from 3.3 to 47 units. Arrows show average end-to-end relocations per 30 min. (TIF) [file pgen.1003956.s002.tif]

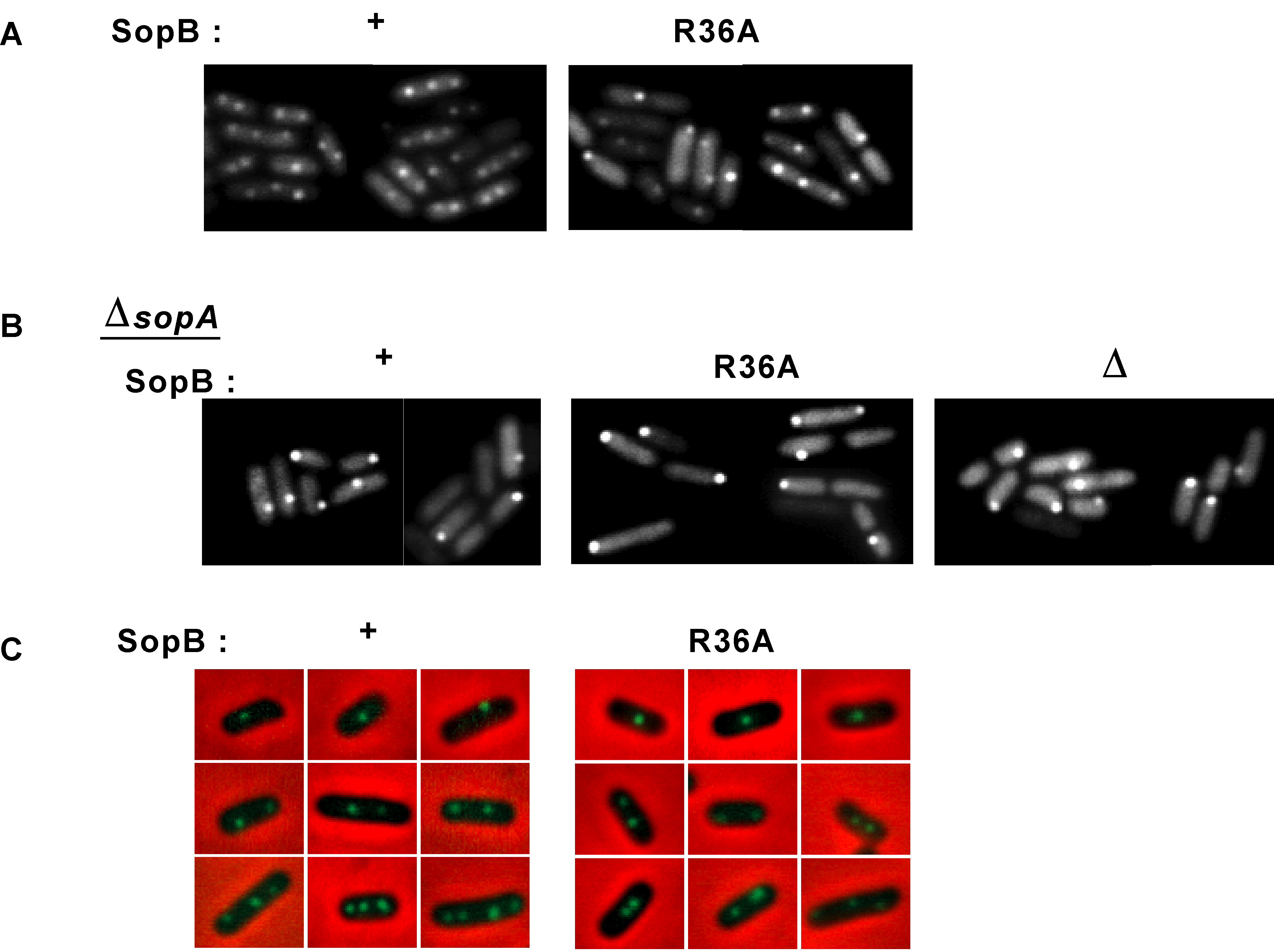

Supplement: Figure S3 — Representative images of cells with labelled mini-F and SopB protein. A. DLT2583 cells carrying mini-F (pDAG848) labelled with TetR::Gfp and producing SopA and wt and R36A mutant SopBs in trans, used to determine mini-F foci per cell (Figure 2A) and focus brightness (Figure 2C). Note that in general the R36A foci are less numerous and brighter. B. DLT2583 cells carrying mini-F (pDAG848) labelled with TetR::Gfp and producing wt and R36A mutant SopBs in trans, used to determine mini-F foci per cell (Figure 2A) and distribution (Figure 2D) in the absence of SopA. Regardless of the sopB allele, foci are generally single, polar and brighter than with SopA present. C. DLT2202 cells carrying mini-F (pDAG209) and producing wt and R36A SopB::mVenus from pJYB259 and 260 respectively. (TIF) [file pgen.1003956.s003.tif]

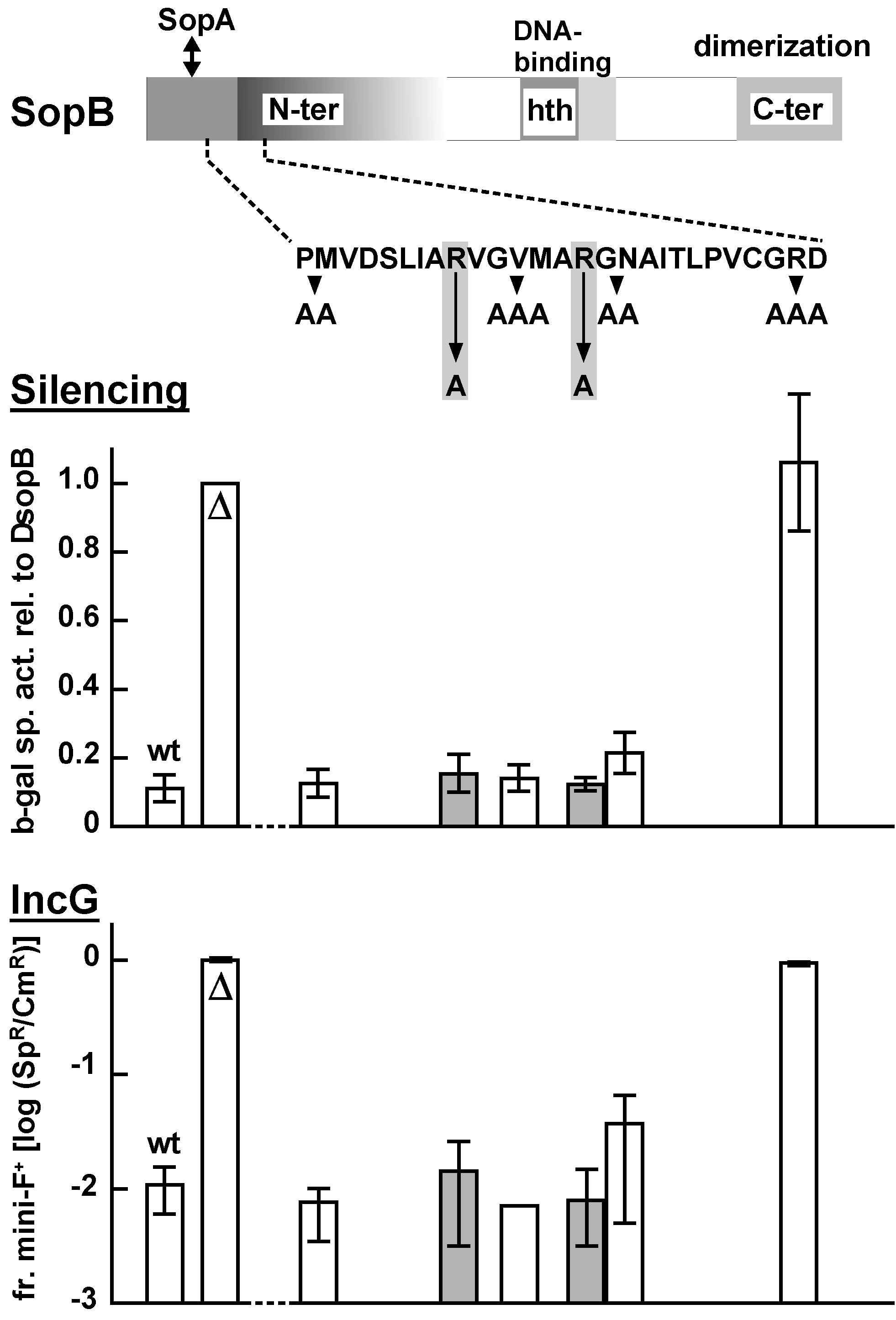

Supplement: Figure S4 — Auto-interaction of mutant SopB proteins. To test whether the sopB R36A and R42A alleles might affect partition by altering the ability of SopB protein to interact with itself (“oligomerize”) we incorporated the two mutants in a separate study aimed at mapping domains of interaction within the SopB N-terminus. The results for these and nearby mutations are presented here. Two indicators of in vivo SopB-SopB interaction were used: silencing of a sopC-proximal promoter, which results from spreading of the specific SopB-sopC complex along adjacent DNA by recruitment of further SopB dimers; and destabilization of a ΔresD mini-F (“IncG incompatibility”), which appears to depend on induction of mini-F pairing/clustering by excess SopB [9]. Silencing of the paadA::lacZ fusion in strain DLT2684 (DLT2067 [9] with gen substituted for cat) was measured by introducing the paraBAD::sopB plasmid pDAG170 [21] and its sopB mutant derivatives, and measuring β-galactosidase specific activity following exponential growth of the resulting strains for about four generations in LB broth supplemented with 0.4% glucose, 20 µg/ml chloramphenicol and 10−4 M arabinose. To confirm the presence of the mutant SopBs at concentrations similar to that of the wt protein, samples of the same cultures were analyzed by electrophoresis in bis-tris denaturing gels (NuPAGE; Invitrogen), where they appear as a light but distinct ∼40 kD band (not shown). Mini-F destabilization was measured by introducing the same plasmids into DLT1900 (DLT2067 without paadA::lacZ) carrying the ΔresD mini-F, pZC209 (SpcR; [28]), growing the resulting strains for about seven generations as above, spreading samples on non-selective LB agar and replica-plating the colonies on LB agar supplemented with 30 µg/ml spectinomycin. The top bar shows the known functional anatomy of SopB. The relevant N-terminal region is magnified to show the double, triple and R-finger mutants analyzed. Silencing data from 4–6 separate determinations [file pgen.1003956.s004.tif]
