# Supplementary material for: Defining the Role of ATP Hydrolysis in Mitotic Segregation of Bacterial Plasmids
Source: PLoS Genet. 2013 Dec 19;9(12):e1003956. doi: 10.1371/journal.pgen.1003956 (PMC3868542; doi:10.1371/journal.pgen.1003956)
Supplement: Table S1 — Effect of the sopB-R36A mutation in trans on mini-F (Δ(sopAB), sopC +) stability. LR - % mini-F lost per generation, average of two or three determinations; SF - stabilization factor (LRΔsopB/LRsopB +/R36A); DsF - destabilization factor (LRsopB R36A/LRsopB +); U-mF – concentration of SopA relative to that in cells carrying wt mini-F (pDAG114), determined independently for each of the strain-growth medium combinations. Corresponding SopA concentrations are shown, as the average values obtained for cells expressing the wt and R36A sopB alleles. * denotes data shown in Figures S1 A and B, other data are from parallel determinations not shown. Because sopAB expression is subject to LacI control in DLT2583 SopA concentrations are lower than in the Δ(lacIZYA) strain, DLT1900; this contributes to the low stabilization factors seen with wt SopB and consequently the lower destabilization factors seen with SopBR36A, relative to those obtained with DLT1900. (DOC) [file pgen.1003956.s005.doc]

**Table S1** Effect of the sopB-R36A mutation *in trans* on mini-F (Δ(*sopAB*), *sopC*^+^) stability.

*ΔsopB* *sopB^+^* *sopB*^R36A^ [SopA]

strain medium LR LR SF LR SF DsF U-mF

DLT1900 min-gly 10.0 0.005 2000 5.0 2 1000 10.0*

min-gly 12.3 0.2 62 0.7 18 3.5 0.6

min-gly- 13.7 0.04 340 0.3 46 7.5 0.9

0.1 µM iptg

DLT2583 MGC 6.2 0.6 10 2.3 2.7 3.8 1.9*

MGC- 6.2 0.3 21 1.6 3.9 5.3 2.5

0.1 µM iptg

LB 1.0 0.003 330 0.15 6.7 50 2.2*

LR - % mini-F lost per generation, average of two or three determinations; SF - stabilization factor (LR_Δ_*_sopB_*/LR*_sopB_*_+/R36A_); DsF - destabilization factor (LR*_sopB_*_R36A_/LR*_sopB_*_+_); U-mF – concentration of SopA relative to that in cells carrying wt mini-F (pDAG114), determined independently for each of the strain-growth medium combinations.

Corresponding SopA concentrations are shown, as the average values obtained for cells expressing the wt and R36A *sopB* alleles. * denotes data shown in Fig. S1 A and B, other data are from parallel determinations not shown. Because *sopAB* expression is subject to LacI control in DLT2583 SopA concentrations are lower than in the Δ(*lacIZYA*) strain, DLT1900; this contributes to the low stabilization factors seen with wt SopB and consequently the lower destabilization factors seen with SopB^R36A^, relative to those obtained with DLT1900.
