# Supplementary material for: Defining the Role of ATP Hydrolysis in Mitotic Segregation of Bacterial Plasmids
Source: PLoS Genet. 2013 Dec 19;9(12):e1003956. doi: 10.1371/journal.pgen.1003956 (PMC3868542; doi:10.1371/journal.pgen.1003956)
Supplement: Table S2 — Copy number of mini-F pDAG209 (relative to pAM238) provided with wt and mutant SopB proteins. DNAs in mini-preps from cells growing exponentially or in stationary phase in LB at 30°C were linearized by digestion with PciI and the fragments quantified by gel-electrophoretic resolution, ethidium bromide staining and fluorescence measurement. Values are averages of two determinations. Fluorescence intensity measurements were corrected for the difference in length of the mini-F (7.6 kb) and the SopB-producing derivatives of pAM138 (6.4 kb), to enable calculation of copy number ratios. (DOC) [file pgen.1003956.s006.doc]

**Table S2.** Copy number of mini-F pDAG209 (relative to pAM238) provided with wt and mutant SopB proteins.

pDAG209 / pAM238

SopB : cells in : exponential stationary

_______________________________________

wt 0.33 0.44

R36A 0.34 0.42

R36K 0.27 0.41

R42A 0.28 0.44

R42K 0.28 0.43

__________________________________________

DNAs in mini-preps from cells growing exponentially or in stationary phase in LB at 30°C were linearized by digestion with *Pci*I and the fragments quantified by gel-electrophoretic resolution, ethidium bromide staining and fluorescence measurement. Values are averages of two determinations. Fluorescence intensity measurements were corrected for the difference in length of the mini-F (7.6 kb) and the SopB-producing derivatives of pAM138 (6.4 kb), to enable calculation of copy number ratios.
