# Supplementary material for: Defining the Role of ATP Hydrolysis in Mitotic Segregation of Bacterial Plasmids
Source: PLoS Genet. 2013 Dec 19;9(12):e1003956. doi: 10.1371/journal.pgen.1003956 (PMC3868542; doi:10.1371/journal.pgen.1003956)
Supplement: Text S1 — Construction of strains carrying Ω (para-tetR::egfp) and ΔlacZΩ (plac-sopA::xfp). (DOC) [file pgen.1003956.s007.doc]

Supporting Information - Text S1

Construction of strains carrying Ω(*para*-*tetR*::*egfp*) and Δ*lacZ*Ω(*plac-sopA*::*xfp*).

Ω(*para*-*tetR*::*egfp*) The *yfp* moiety of the *tetR*::*yfp* fusion in pFX234 (equivalent to pLAU18; Lau et al, 2003) was replaced by gfp (corresponding to amino acid changes F64L, G65T, L68V, A72S and Y203T) to give pDAG729. The *frt-cat-frt* fragment was inserted as below (Figure si1) to form pDAG739. Oligonucleotides araC1 5'-CTCGTCCCTGATTTTT CACCACCCCCTGACCGCGAATGGTGAGATTGAGA and araD1 5'-GTTTCGATGCT AACCACGACCATATCGTCAGCGGTCATGACGCTGTAATCCTATTATGAATATCCTCCTTAG (chromosome target sequences underlined) were used to amplify *frt-cat-frt* with *tetR::gfp* as shown, and the product integrated by recombineering (Datsenko & Wanner, 2000) into DLT2235 (DLT2202 carrying λ*imm*^434^cIind^-^-*kan*-*lacOP*::*sopA*^+^*B*^+^) using pKD46, to create DLT2571 (Figure si2).


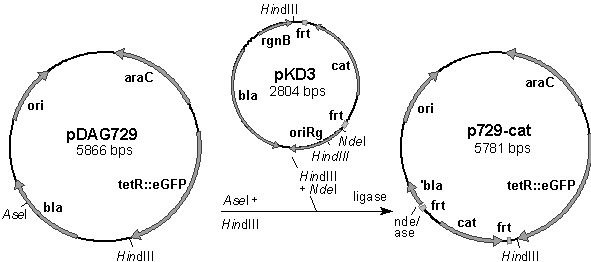


**pDAG739**

**araC1**

**araD1**

Figure si1.


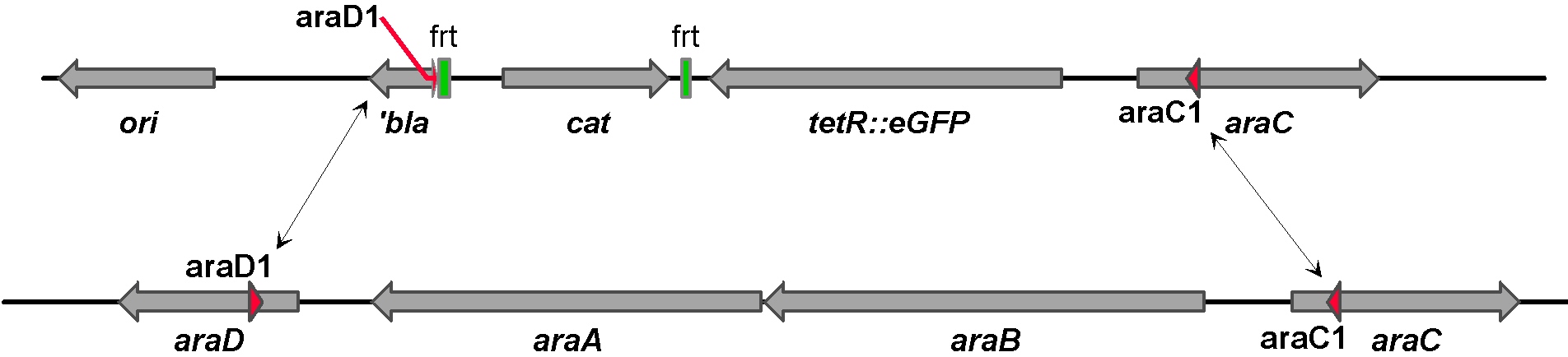


Figure si2.

The Δ(*araBAD'*) Ω(*para*-*tetR*::*egfp*) was shifted to DLT2202 by P1 transduction, giving DLT2573, and the *frt-cat-frt* element removed by FLP-mediated excision using pCP20 (Datsenko & Wanner, 2000), yielding strain DLT2583 (see Table 1).


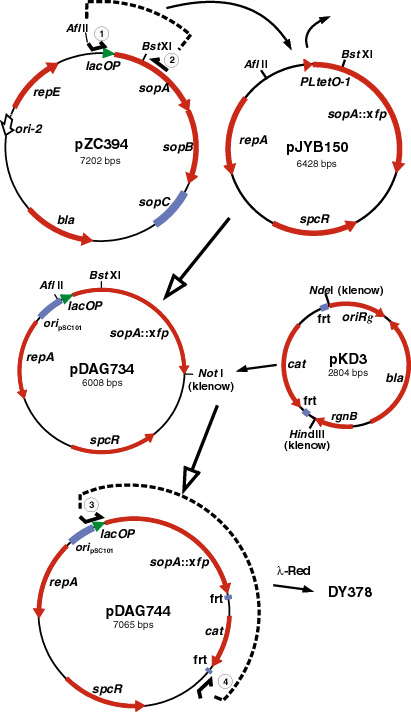
Δ*lacZ*Ω(*plac-sopA*::*xfp*) The *sopA::xfp* fusion, constructed as described in Castaing et al (2008), was placed under control of the wt *lac* promoter from pZC394 (Yates et al, 1999) to create pDAG734, and the FLP-excisable chloramphenicol-resistance gene module then inserted adjacent to it to form pDAG744., as shown in the figure. Oligonucleotides tailed by *lac* operon sequences corresponding to nucleotides 365671-365597 and 362393-362452 of the *E.coli* chromosome were used to amplify the *sopA::xfp-cat* segment which was then integrated into the chromosome of the λ-Red induction strain, DY378, yielding DLT2629

The segment was moved by P1 transduction to DLT1900 and DLT2583 (Table 1), and the *cat* module then removed by FLP-mediated excision using pCP20 (Datsenko & Wanner, 2000), to yield the MC1061- and MG1655-based strains DLT2687 and DLT2740 respectively (see Table 1).

Oligonucleotide primer sequences, indicated by circled numbers 1-4 in Figure si3, are given below; chromosome sequences are underlined.

Figure si3

1 - pSCoriLacO3

5'-CCCTAAAGGCTTAAGTAGCACCCTCGCAAGCTCGGGCAAATCGCTGAATCTG GAAAGCGGGCAGTGAGCGCAACGCAATT

2 - SopA520c

5'-GCTTTATTGCATAAGTGACATCGTC

3 - Plac734cat_Ig

5'-**GACTGGAAAGCGGGCAGTGAGCGCAACGCAATTAATGTGAGTTAGCTC ACTCATTAGGCACCCCAGGCTTTACAC**

4 - gapZYIg

5'-**AAATAGTACATAATGGATTTCCTTACGCGAAATACGGGCAGACATGGCC TGCCCGGTTATATTGTGTAGGCTGGAGCTGC**

References

Lau, I.F., Filipe S.R., Søballe B., Økstad, O-A., F-X. Barre & Sherratt, D.J. (2003) Spatial and temporal organization of replicating *Escherichia coli* chromosomes. *Mol. Microbiol.* 49: 731-743

Datsenko, K.A. & Wanner, B.L. (2000) One-step inactivation of chromosomal genes in *Escherichia coli* K-12 using PCR products. *Proc Natl Acad Sci USA* **97:** 6640–6645

Castaing, J.P., Bouet, J.Y. & Lane, D. (2008) F plasmid partition depends on interaction of SopA with non-specific DNA. *Mol Microbiol* **70:** 1000-1011

Yates, P., Lane, D. & Biek, D.P. (1999) The F plasmid centromere, *sopC*, is required for full repression of the *sopAB* operon. *J Mol Biol* **290:** 627-638
